# Predicting the fMRI Signal Fluctuation with Recurrent Neural Networks Trained on Vascular Network Dynamics

**DOI:** 10.1093/cercor/bhaa260

**Published:** 2020-09-17

**Authors:** Filip Sobczak, Yi He, Terrence J Sejnowski, Xin Yu

**Affiliations:** Translational Neuroimaging and Neural Control Group, High Field Magnetic Resonance Department, Max Planck Institute for Biological Cybernetics, 72076 Tuebingen, Germany; Graduate Training Centre of Neuroscience, International Max Planck Research School, University of Tuebingen, 72074 Tuebingen, Germany; Translational Neuroimaging and Neural Control Group, High Field Magnetic Resonance Department, Max Planck Institute for Biological Cybernetics, 72076 Tuebingen, Germany; Danish Research Centre for Magnetic Resonance, 2650, Hvidovre, Denmark; Howard Hughes Medical Institute, Computational Neurobiology Laboratory, Salk Institute for Biological Studies, La Jolla, CA 92037, USA; Division of Biological Sciences, University of California, San Diego, La Jolla, CA 92093, USA; Translational Neuroimaging and Neural Control Group, High Field Magnetic Resonance Department, Max Planck Institute for Biological Cybernetics, 72076 Tuebingen, Germany; Athinoula A. Martinos Center for Biomedical Imaging, Massachusetts General Hospital and Harvard Medical School, Charlestown, MA 02129, USA

**Keywords:** default mode network, machine learning, resting state, single-vessel, slow oscillation

## Abstract

Resting-state functional MRI (rs-fMRI) studies have revealed specific low-frequency hemodynamic signal fluctuations (<0.1 Hz) in the brain, which could be related to neuronal oscillations through the neurovascular coupling mechanism. Given the vascular origin of the fMRI signal, it remains challenging to separate the neural correlates of global rs-fMRI signal fluctuations from other confounding sources. However, the slow-oscillation detected from individual vessels by single-vessel fMRI presents strong correlation to neural oscillations. Here, we use recurrent neural networks (RNNs) to predict the future temporal evolution of the rs-fMRI slow oscillation from both rodent and human brains. The RNNs trained with vessel-specific rs-fMRI signals encode the unique brain oscillatory dynamic feature, presenting more effective prediction than the conventional autoregressive model. This RNN-based predictive modeling of rs-fMRI datasets from the Human Connectome Project (HCP) reveals brain state-specific characteristics, demonstrating an inverse relationship between the global rs-fMRI signal fluctuation with the internal default-mode network (DMN) correlation. The RNN prediction method presents a unique data-driven encoding scheme to specify potential brain state differences based on the global fMRI signal fluctuation, but not solely dependent on the global variance.

## Introduction

Neural oscillations have been extensively studied in both animal and human brains from cellular to systems levels (Steriade 2001; Buzsáki and Draguhn 2004; Masimore et al. 2004; Muller et al. 2018). Power profiles of EEG signals, as well as slow cortical potentials (SCP), exhibit a slow oscillation feature (<1 Hz), which is related to brain states mediating memory, cognition and task-specific behaviors (Birbaumer et al. 1990; Elbert 1993; He and Raichle 2009). Resting-state functional MRI (rs-fMRI) studies have revealed low-frequency hemodynamic signal fluctuations (<0.1 Hz) (Biswal et al. 1995; Biswal et al. 1997; Cordes et al. 2001; Fukunaga et al. 2006), which have been confirmed by intrinsic optical imaging (Kleinfeld et al. 1998), laser-doppler-flowmetry (Golanov et al. 1994), and near-infrared spectroscopy (Obrig et al. 2000). In particular, specific spatial correlation patterns can be observed in the slow oscillation of the rs-fMRI signal, e.g., the default-mode network (DMN) (Raichle et al. 2001; Greicius et al. 2003; Hampson et al. 2006). Concurrent fMRI and electrophysiology studies have shown a correlation of the fMRI signal fluctuation with the EEG signal power profile and SCP low-frequency oscillations, which are candidates for neural correlates of the rs-fMRI signal (Logothetis et al. 2001; Goldman et al. 2002; He et al. 2008; Shmuel and Leopold 2008; Scholvinck et al. 2010; Pan et al. 2013; Fultz et al. 2019). In addition, the slow oscillation of rs-fMRI and hemodynamic signals from vessels are highly correlated to simultaneously acquired intracellular Ca^2+^ signal fluctuations in rodents (Du et al. 2014; Ma et al. 2016; Schwalm et al. 2017; He et al. 2018; Chen et al. 2020).

Efforts have been made to interpret the functional indications of rs-fMRI spatial correlation patterns, including research revealing a rich repertoire of states and their transitions that constitute the rs-fMRI signal (Chang and Glover 2010; Handwerker et al. 2012; Hutchison et al. 2013; Liu et al. 2013; Liu and Duyn 2013; Hansen et al. 2015; Karahanoglu and Van De Ville 2015; Liang et al. 2015; Chen et al. 2016; Vidaurre et al. 2017; Yousefi et al. 2018), as well as arousal state-dependent global fMRI signal fluctuation studies (Chang et al. 2016; Turchi et al. 2018; Wang et al. 2018). Because of the high variability in different dynamic states, physiological and non-physiological confounding factors also contribute to the rs-fMRI low-frequency oscillation (Birn et al. 2006; Caballero-Gaudes and Reynolds 2017; Pais-Roldán et al. 2018; Tong et al. 2019). In particular, global fMRI signal fluctuations are one of the most controversial oscillatory features to be linked to dynamic brain signals (Fox et al. 2009; Murphy et al. 2009; Hahamy et al. 2014; Murphy and Fox 2017; Power et al. 2017; Billings and Keilholz 2018; Liu et al. 2018; Xu et al. 2018). Efforts have been made to disambiguate the global and vascular signals, to separate the physiological components of the global signal (Glasser et al. 2018) and to remove those components e.g., by using the signal from the white-matter tract as a nuisance regressor (Behzadi et al. 2007; Chang and Glover 2009). Interestingly, the global signal has been tied to behavioral traits (Li et al. 2019) and vigilance (Wong et al. 2013; Wong et al. 2016) of scanned subjects and the global signal fluctuation has been tied to the switching of whole brain spatial patterns (Gutierrez-Barragan et al. 2019). Moreover, simultaneous fMRI and EEG studies in the monkey brain demonstrate a strong linkage of brain state changes to the global rs-fMRI signal fluctuations (Scholvinck et al. 2010). This phenomenon has been observed at the level of single-vessel fMRI dynamic mapping with concurrent calcium recordings (Yu et al. 2016; He et al. 2018; Chen et al. 2019), showing stronger neural correlation from vessel voxels than parenchyma voxels given the highly deoxygen-hemoglobin-based T2^*^-weighted contrast-to-noise ratio (CNR) changes (He et al. 2018). This highly coherent vessel-specific fMRI signal fluctuation is a direct signal source that is closely linked to global brain state changes. Here, we applied the artificial state-encoding recurrent neural network system in a prediction scheme to better model the brain state-specific coherent oscillatory features from the vessel voxels.

Recurrent neural networks (RNNs) provide a computational framework for temporally predicting dynamic brain signals. RNNs, through interactions of recurrently connected simple computational nodes (neurons), encode temporal patterns of input signals, i.e., the vessel specific rs-fMRI signals, into internal states. These states are then decoded to generate predictions e.g., using linear weighting. Two example RNN architectures both employing gating mechanisms and trained through backpropagating errors (Linnainmaa 1976; Rumelhart et al. 1988) are the gated recurrent unit (GRU) (Cho et al. 2014) and long short-term memory (LSTM) (Hochreiter and Schmidhuber 1997; Gers et al. 2003) networks. These RNNs have been applied to fMRI data to e.g., model hemodynamic response functions (Güçlü and van Gerven 2017), decode task properties (Li and Fan 2018), identify individuals (Chen and Hu 2018) and integrate behavioral and neuroimaging data in a decision task (Dezfouli et al. 2018). In particular, the artificial neural networks have been used to depict dynamic brain signals over a range of time scales and contexts (Plis et al. 2014; Yamins et al. 2014; Barrett et al. 2018; Hjelm et al. 2018; Wen et al. 2018).

In the present study, GRUs were trained to predict the slow oscillation dynamic changes of the rs-fMRI signal from both rat and human brains. Based on previous single-vessel fMRI studies (He et al. 2018), vessel-specific fMRI signals were used as training data to extract highly correlated neuronal oscillatory temporal features with varied noise profiles. Given the significantly reduced auto-regression features of the slow oscillation after a 10 s lag time, we trained the RNNs to predict the temporal evolution of slow oscillations with the 10 s interval into the future. The trained networks encoded unique temporal dynamic features of the rs-fMRI signal, enabling the differentiation of the global fMRI signal fluctuation from the DMN-specific temporal dynamic patterns in the Human Connectome Project (HCP) data (Van Essen et al. 2012). In particular, in contrast to the global variance analysis, the RNN-based prediction presents a linear association to the strength of DMN-specific network correlation indicating a unique data-driven encoding scheme to specify brain state differences.

## Materials and Methods

### GRU

Gated recurrent unit (GRU) (Cho et al. 2014) networks are an RNN architecture designed to tackle the vanishing and exploding gradient problems, which prevented effective learning in networks trained using backpropagation. They introduce gating mechanisms that control the flow of information into and out of the GRU units and allow the network to capture dependencies at different time scales in the processed data. The GRU encodes each element of the input single-vessel sequence $\boldsymbol{x}$ into a hidden state vector $\boldsymbol{h}(t)$ by computing the following functions:

$$ \boldsymbol{r}(t)=\sigma \left({\boldsymbol{W}}_{ir}x(t)+{\boldsymbol{b}}_{ir}+{\boldsymbol{W}}_{hr}\boldsymbol{h}\left(t-1\right)+{\boldsymbol{b}}_{hr}\right) $$

$$ \boldsymbol{z}(t)=\sigma \left({\boldsymbol{W}}_{iz}x(t)+{\boldsymbol{b}}_{iz}+{\boldsymbol{W}}_{hz}\boldsymbol{h}\left(t-1\right)+{\boldsymbol{b}}_{hz}\right) $$

$$ \boldsymbol{n}(t)=\tanh \left({\boldsymbol{W}}_{in}x(t)+{\boldsymbol{b}}_{in}+\boldsymbol{r}(t)\begin{array}{c}\!\!\!\odot\!\!\!\!\!\end{array} \left({\boldsymbol{W}}_{hn}\boldsymbol{h}\left(t-1\right)+{\boldsymbol{b}}_{hn}\right)\right) $$

$$ \boldsymbol{h}(t)=\left(1-\boldsymbol{z}(t)\right)\begin{array}{c}\!\!\!\!\odot\!\!\!\!\end{array} \boldsymbol{n}(t)+\boldsymbol{z}(t)\begin{array}{c}\!\!\!\odot\!\!\!\end{array} \boldsymbol{h}\left(t-1\right) $$

where $\sigma \big(\big),\mathit{\tanh}\ \big(\big)$ are the sigmoid and hyperbolic tangent functions, $\boldsymbol{r},\boldsymbol{z},\boldsymbol{n}$ are the reset, update and new gates, $\boldsymbol{W}$ are matrices connecting the gates, inputs and hidden states, $\boldsymbol{b}$ are bias vectors and $\odot$ is the elementwise product. A linear readout was used to generate the prediction based on the state vector:

$$ y(\mathit{\mathsf{t}})={\boldsymbol{\mathsf{w}}}_{\mathit{\mathsf{out}}}\boldsymbol{h}(\mathit{\mathsf{t}}). $$

The networks were trained in PyTorch (Paszke et al. 2019) and cross-validated across trials. The hyperparameters were found with Bayesian optimization using the tree of Parzen estimators algorithm (Hyperopt toolbox, n = 200) (Bergstra et al. 2011; Bergstra et al. 2013). The optimized hyperparameters have been described in Table 1.

**Table 1:** Optimized GRU hyperparameters

Parameter name	Description	Range	Final value (rat | human)
Number of layers	Multiple layers of each of the recurrent units could be stacked on top of each other.	[1; 5]	2 | 1
Hidden size	Size of the hidden state vector.	[10; 500]	290 | 88
Loss function	As the Pearson correlation coefficient (CC) was the final evaluation metric of networks’ performance, it could be used as the cost function instead of the mean squared error (MSE) loss.	[MSE, CC, MSE and CC]	CC | CC
Learning rate	A parameter defining the rate at which network weights were updated during training.	[10^−5^; 1]	0.001 | 0.00121
L2	Strength of the L2 weight regularization.	[0; 10]	0.0003 | 0.0221
Gradient clipping	Gradient clipping (Pascanu et al. 2013) limits the magnitude of the gradient to a specified value.	[yes; no]	no | no
Dropout	In the case of using a multi-layer RNN, dropout (Srivastava et al. 2014) could be set.	[0; 0.2]	0.128 |—
Residual connection	Employing a residual connection i.e., feeding the input directly to the linear readout alongside the RNN’s hidden state.	[yes; no]	yes | no
Batch size	The number of single-vessel time courses processed by the network in the training stage before each weight update.	[3; 32]	22 | 10
Number of epochs	How many times the network processed the whole training dataset during training.	[1; 100]	87 | 69
Washout time	The number of input signals’ time points used to drive the network into a state that is specific to a given input. These time points are not used for readout training and prediction.	Fixed	250 | 250

### ARMAX

The autoregressive-moving-average model with exogenous inputs (ARMAX) (Whittle 1951) was used as a comparative prediction method. ARMAX aims to model a time series using autoregressive, moving-average and exogenous input terms. This is depicted in the equation:

$$\begin{align*} & y(t)+{a}_1y(t-1)+\dots +{a}_{n_a}y(t-{n}_a)={b}_1u(t-{n}_k)+\dots +{b}_{n_b}u\\ & (t-{n}_k-{n}_b+1)+{c}_1e(t-1)+\dots +{c}_{n_c}e(t-{n}_c)+e(t), \end{align*}$$

where $y(t)$ is the model’s output at time $t$; $u(t)$ is the exogenous input at time $t$; $e(t)$ is the noise term at time $t$; ${n}_a,{n}_b,{n}_c$ are the numbers of model’s past outputs, inputs and error terms that influence the current output; ${n}_k$ is the delay after which the inputs influence the output; ${a}_i,{b}_i,{c}_i$ are estimated model coefficients. To match the 10 s prediction scheme ${n}_k$ was set to 10 and the raw inputs and slow oscillation outputs were not shifted. An extensive grid search was performed to find the ${n}_a,{n}_b,{n}_c$ values that led to the best predictions. All combinations of ${n}_a,{n}_b,{n}_c$ values ranging from 1 to 50 with a step of 1 and from 1 to 150 with a step of 5 were evaluated to estimate the model’s coefficients ${a}_i,{b}_i,{c}_i$. Exactly the same data as in GRU’s case were used for training and evaluation and the best set of ${n}_a,{n}_b,{n}_c$ values was also found through cross-validation. MATLAB *armax* and *forecast* functions were used to find the coefficient values and evaluate the models. The autoregressive model with exogenous input (ARX) and the autoregressive-integrated-moving-average model with exogenous inputs (ARIMAX) were also tested but yielded worse performances, hence are not reported.

### Experimental Procedures

All experimental procedures were approved by the Animal Protection Committee of Tuebingen (Regierungsprasidium Tuebingen) and performed in accordance with the guidelines. All human subject experiments follow the guidelines of the regulation procedure in the Max Planck Institute, and the informed consents were obtained from all human volunteers. Single-vessel fMRI data acquired from 6 rats and 6 human subjects have been previously published (He et al. 2018). The rats were imaged under alpha-chloralose anesthesia. For details related to the experimental procedures refer to (Yu et al. 2010; He et al. 2018).

### Rat MRI Data Acquisition

The measurements have been performed using a 14.1 T/26 cm horizontal bore magnet (Magnex) interfaced with an Avance III console (Bruker). To acquire the images a 6 mm (diameter) transceiver surface coil was used.

### bSSFP rs-fMRI

The balanced steady-state free precession (bSSFP) sequence was used to acquire 2–5 trials of single-slice Blood-oxygen-level-dependent (BOLD) rs-fMRI for every rat. Each run had a length of 15 minutes with a one slice repetition time of 1 s. The bSSFP parameters were: echo time (TE) = 3.9 ms; repetition time (TR) = 7.8 ms; flip angle = 12°; matrix = 96 × 128; field of view (FOV) = 9.6 × 12.8 mm; slice thickness = 400 μm; in-plane resolution = 100 × 100 μm^2^.

### MGE A-V Map Acquisition in Rats

To detect individual blood vessels a 2D multi-gradient-echo (MGE) sequence was used. The sequence parameters were: TR = 50 ms; TE = 2.5, 5, 7.5, 10, 12.5 and 15 ms; flip angle = 40°; matrix = 192 × 192; in-plane resolution = 50 × 50 μm^2^; slice thickness = 500 μm. The second up to the fifth echoes of the MGE images were averaged to create arteriole-venule (A-V) maps (Yu et al. 2016). The A-V maps enable identifying venule voxels as dark dots due to the fast T2^*^ decay and arteriole voxels as bright dots because of the in-flow effect.

### Human MRI Data Acquisition

Data from six healthy adult subjects (male, n = 3; female, n = 3; age: 20–35 years) were acquired using a 3-T Siemens Prisma with a 20-channel receive head coil. BOLD rs-fMRI measurements were performed using an echo-planar imaging (EPI) sequence with: TR = 1000 ms; TE = 29 ms; flip angle = 60°; matrix = 121 × 119; in-plane resolution = 840 μm × 840 μm; 9 slices with thicknesses of 1.5 mm. Image acquisition was accelerated with parallel imaging (GRAPPA factor: 3) and partial Fourier (6/8). Subjects had their eyes closed during each 15 minute trial. Respiration and pulse oximetry were simultaneously monitored using the Siemens physiologic Monitoring Unit.

### Data Preprocessing

All data preprocessing was done using MATLAB and the Analysis of Functional Neuro Images (AFNI) software package (Cox 1996). The functional data were aligned with the A-V map using the mean bSSFP template and the *3dTagAlign* AFNI function with 10 tags located in the venule voxels. Other details of the preprocessing procedure are reported in a previous study (Zhang et al. 2012). No spatial smoothing was done at any point.

### Localization of Individual Veins

To localize venule voxels in A-V maps, local statistics analysis and thresholding were performed using AFNI. First, for each voxel, the minimum value in a 1 voxel-wide rectangular neighborhood was found. Then, the resulting image was filtered with a 10 voxel rectangular rank filter and divided by the size of the filter. Finally, the image was thresholded to create a mask with vein locations. For human data, the mean of EPI time series was used instead of the A-V map.

### ICA Identification of Vascular Slow Oscillations

To extract signals only from vessels exhibiting strong slow oscillations an additional independent components analysis (ICA)-based mask was combined with the described above vessel localization method. The functional rs-fMRI data were processed using the Group ICA of fMRI Toolbox (GIFT, http://mialab.mrn.org/software/gift) in MATLAB. First, principal component analysis (PCA) was employed to reduce the dimensionality of the data. PCA output was used to find 10 independent components and their spatial maps using spatial Infomax ICA (Bell and Sejnowski 1995). If a component exhibiting slow oscillations predominantly in individual vessels had been found, it was thresholded and used together with the vascular mask to identify vessels of interest and extract their signals.

### Frequency Normalization

To normalize the data, power density estimates of signals’ high-frequency components were used. Every time course had its mean removed and was divided by the mean power spectral density estimate (PSD) of its frequency components higher than 0.2 Hz. The 0.2 Hz point was chosen, as above this value spectra of extracted signals were centered on a horizontal, non-decaying line. Performing the division brought the mean PSD of high-frequency components to a common unit baseline for all signals.

This allowed to better compensate for different signal strengths across trials than when scaling the data using minimal and maximal values. Additionally, the relative strength of flatter signals and those exhibiting stronger low-frequency oscillations was better preserved when compared to variance normalization. Ultimately it also improved prediction performance.

### Power Spectrum Analysis

The spectral analysis was performed in MATLAB. To compute the PSDs of utilized signals we employed Welch’s method (Welch 1967) with the following parameters: 1024 discrete Fourier transform points; Hann window of length 128; 50% overlap.

### Filtering

To obtain target signals, single-vessel time courses were bandpass filtered in MATLAB using *butter* and *filtfilt* functions. The frequency bands (0.01–0.1 for human and 0.01–0.05 for rat data) were chosen based on the PSD curves of single-vessel and ICA time courses.

### Surrogate Data Generation

Surrogate data methods are primarily used to measure the degree of nonlinearity of a time series (Theiler et al. 1992). They allow creating artificial time courses that preserve basic statistics of original data like the mean, variance and autocorrelation structure. In this study, Fourier based surrogate signals were generated for each single-vessel time course using the iterative amplitude adjusted Fourier transform (IAAFT) algorithm (Schreiber and Schmitz 1996).

To create a surrogate control, a list of a signal’s amplitude-sorted values and the complex magnitudes of its Fourier frequency decomposition need to be saved. First, the original signal is randomly reordered. The complex magnitudes of the shuffled signal are replaced by the stored values of the original signal with the new phases being kept. This changes the amplitude distribution. To compensate for this, the new signal’s sorted values are assigned values from the stored ordered amplitude distribution of the source signal (the new signal is only sorted for the assignment, its order is restored afterwards). In turn, matching the amplitudes modifies the spectrum, so the complex magnitude and amplitude matching steps are repeated and the modified phases of the resulting signal are kept through iterations.

The iteratively generated signals had the same amplitude distribution as the source data and extremely similar amplitudes of the power spectrum. However, the phases of their complex Fourier components were randomized.

### Principal Component Analysis of GRU Hidden States

We used the MATLAB *pca* function to apply PCA to the network’s hidden states and generate PCA time courses.

### Sliding Window Score Signals

For each time point we computed the correlation between the predicted and target single-vessel signal in 30 s windows. The first and last 15 values of each sliding window signal were based on shorter windows due to the window extending beyond available data.

### HCP Data—Preprocessing

Data from 4012 15-minute sessions of rs-fMRI acquired by the Human Connectome Project (HCP) (Van Essen et al. 2012) were used to extract V1 signals and compute whole-brain correlation maps. The data set was preprocessed (Glasser et al. 2013; Smith et al. 2013), had artifacts removed via ICA + FIX (Griffanti et al. 2014; Salimi-Khorshidi et al. 2014) and was registered to a common space (Robinson et al. 2014; Glasser et al. 2016) by the HCP. The data were resampled from the original 0.72 s sampling rate to match the 1 s TR of our in-house datasets.

### HCP Data—ROI Signal Extraction

The multi-modal cortical parcellations (Glasser et al. 2016) was used to extract 180 region of interest (ROI) signals per hemisphere. The DMN ROI was based on the DMN ROI specified in Yeo et al. (Yeo et al. 2011). Subcortical structures were extracted using the Connectome Workbench (Marcus et al. 2011). The global signal was computed by averaging signals of all cortical voxels.

### HCP Data—ICA Parcellations

ICA spatial maps and their corresponding time courses for each rs-fMRI session were obtained from the S1200 Extensively Processed fMRI Data released by HCP. The spatial maps are based on group-PCA results generated using MIGP (MELODIC’s Incremental Group-PCA) (Smith et al. 2014). Spatial ICA was applied to the group-PCA output using FSL’s MELODIC tool (Hyvarinen 1999; Beckmann and Smith 2004). To derive component-specific time courses for each session, the spatial maps were regressed against the rs-fMRI data (Filippini et al. 2009). In this work, we used results from the 15-component decomposition. 4012 rs-fMRI sessions had the ICA results available.

### Spatial Correlation Difference Maps Generation

To create a correlation map for one session, the time course of either the V1 ROI, DMN ROI, cortical global mean, DMN ICA or V1 ICA served as the seed which was correlated with all voxel time courses in that session. To generate the difference maps, individual maps of selected sessions were group averaged and subtracted.

### Intrinsic DMN Correlation

Intrinsic DMN correlation in an individual trial was computed as the average correlation between the DMN ICA time course and all individual DMN ROI voxel signals.

### Cross-Correlation

MATLAB *xcorr* and *zscore* functions were used to compute cross-correlation. Lag times were computed between predictions and desired outputs. Positive lags correspond to delayed predictions and negative lags to too early predictions.

### Correlation Matrix Spectral Reordering

To change the order of matrix entries so that ROIs with similar whole-brain correlation patterns were clustered together, a Laplacian-based spectral reordering method was used (Barnard et al. 1995).

### Statistical Tests

The statistical significance of the difference between real/surrogate and GRU/ARMAX prediction scores was verified using a paired t-test (MATLAB *ttest* function). To determine differences between seed-based correlation maps and PSDs two-sample t-tests were applied (MATLAB *ttest2* function). Fisher’s z-transform has been applied to all correlation values before conducting statistical tests. The results have been controlled for false discovery rate with adjustment (Benjamini and Hochberg 1995; Yekutieli and Benjamini 1997). P values < 0.05 were considered statistically significant.

## Results

Two datasets were used in our study, one from rats and another from humans. First, we trained a GRU to encode temporal dynamics of BOLD-fMRI signals from vessel voxels in anesthetized rat brains to estimate the prediction efficiency. Second, we trained another GRU to predict the slow oscillation of fMRI signals from occipital lobe sulcus veins of awake human subjects and applied the GRU trained on human data to classify the brain-state changes from rs-fMRI data acquired by the Human Connectome Project (HCP). We compared the GRU results with the predictions of autoregressive moving average with exogenous input modeling (ARMAX) (Whittle 1951; Box 1976). Lastly, we specified the RNN prediction-based DMN activity classification of the HCP datasets, showing a unique brain state encoding scheme, different from the global variance-based approach.

### Extracting Slow Oscillatory Features of the Single-Vessel fMRI Signal from Rat Brains

**Figure 1:**
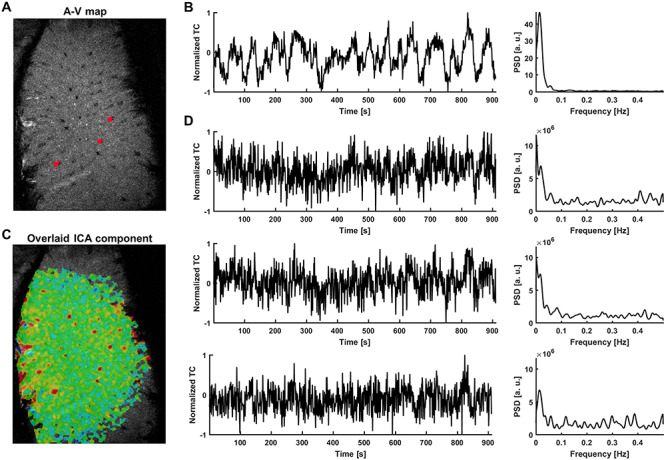
Extraction of signals from single venules exhibiting strong slow fluctuations—rat. (A) The A-V map enables localization of single venules (dark dots) in the rat somatosensory cortex (red—3 vessel masks; plotted in D. (B) Time course of the slowly changing ICA component shaping vascular dynamics and its PSD. (C) The corresponding ICA spatial map highlights the presence of slow fluctuations predominantly in veins. (D) Examples of extracted vascular time courses selected for further processing (marked as red dots on the A-V map in A along with their PSDs. The ICA component is present in the signals, but the noise level is much higher and individual differences are clearly visible.

We used recordings obtained from the balanced steady-state free precession (bSSFP) sequence (Scheffler and Lehnhardt 2003) on single-vessel fMRI data from anesthetized rats (He et al. 2018). Arteriole-venule (A-V) maps based on the multi-gradient-echo (MGE) sequence were acquired to localize individual venules penetrating the cortex, which were shown as dark dots due to the fast T2^*^ decay of the deoxygenated blood (Fig. 1*A*) (Yu et al. 2016). After registering functional data with the A-V map, fMRI time courses from individual venules were extracted and analyzed using independent component analysis (ICA) (Bell and Sejnowski 1995; Mckeown et al. 1998; Calhoun et al. 2009). Figure 1*B* shows the time series of the largest ICA component, which is dominated by the low frequency fluctuation (<0.1 Hz). The superposition of this ICA component with the single-vessel fMRI signal fluctuation on the A-V map overlapped with venule-dominated patterns (Fig. 1*C*). Figure 1*D* shows the raw bSSFP-fMRI signal fluctuation from three venules, as well as their power spectral density (PSD) plots. These data presented highly coherent oscillatory features of single-vessel fMRI signals, which can be used as a training set. It is important to note that this coherent oscillation of vessel-specific rs-fMRI signals is strongly correlated to the concurrent calcium transients, showing brain state-dependent dynamic fluctuation (He et al. 2018).

### Supervised Training of the GRU-Based Prediction of the fMRI Slow Oscillation

**Figure 2:**
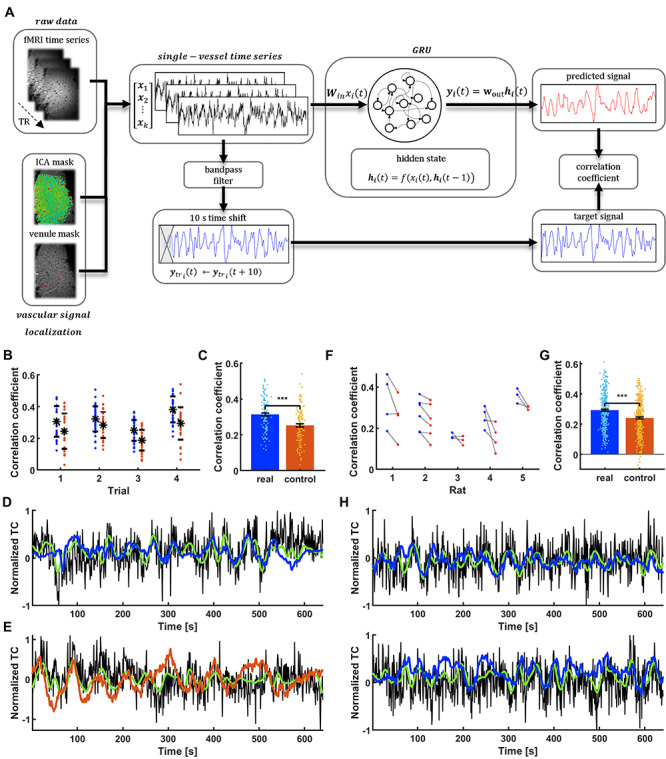
GRU prediction of the spontaneous slow fluctuation of rat vascular dynamics. (A) Prediction system operation pipeline. Raw vascular data are extracted from fMRI data using venule and ICA masks and are fed into the GRU. They are also bandpass filtered and shifted by 10 s to become target outputs of the network. The reservoir encodes the temporal dynamics of input signals into state vectors. The decoder interprets these states and generates a prediction of the slow fluctuation’s value 10 s ahead. After generating the full predicted time series, it is compared with the target output using Pearson’s correlation coefficient. (B) Prediction scores of all the signals extracted from a single rat (blue dots) ordered by trials. Real data are matched with controls (red dots) for every vessel. Black dots show mean scores across trials and bars are SD values. (C) Significantly higher mean of training rat real data prediction scores (CC = 0.31 ± 0.01 SEM) compared to controls (CC = 0.25 ± 0.01 SEM; paired-sample t-test, *P* = 3.7^*^10^–10^). (D) The signal from a single vessel with the best prediction score (CC = 0.51, t_lag_ = −1 s; black—raw data, green—target signal, blue—network prediction). (E) Surrogate signal created to match the real vascular signal shown in D (CC = 0.41, t_lag_ = −4 s; black—raw data, green—target, red—network prediction). (F) Mean prediction scores for trials extracted from five rats (blue) and their corresponding controls (red). (G) Significantly higher mean of different rats’ real data prediction scores (CC = 0.29 ± 0.01 SEM) than controls (CC = 0.24 ± 0.01 SEM; paired-sample t-test, *P* = 6.4^*^10^–24^). (H) Predictions of single-vessel signals from two different rats (v_1_, CC = 0.51, t_lag_ = 0 s; v_2_, CC = 0.52, t_lag_ = 0 s).

Figure 2*A* illustrates the data-driven training scheme for the RNN-based prediction of the rs-fMRI signal fluctuation. The single-vessel fMRI signals showing a strong slow oscillatory correlation (Fig. 1) were used as input time series for the supervised training. The targets of the output were bandpass-filtered fMRI signals from the voxels of the same vessel with a 10 s time shift. The 10 s time shift was selected as the autocorrelation of both rat and human signals is largely reduced at the 10 s lag (Supplementary Fig. 1). Pearson correlation analysis was performed to estimate the correlation coefficient (CC) between GRU’s output predictions and the filtered target signals, to measure the RNN’s performance. We used Bayesian optimization and cross validation to find the set of hyperparameter values that produced the best performing network, see details in the Methods section).

### GRU-Based Single-Vessel fMRI Slow Oscillation Prediction in Anesthetized Rats

We first illustrate the predictive capacity of the trained GRU by analyzing correlation coefficients across all cross-validation tests. Figure 2*B* demonstrates the CC of the slow oscillation prediction of all vessels from a representative rat. For each vessel, we generated a surrogate control time course that mimicked the frequency power profile of the fMRI signal. To differentiate the control dataset from true brain dynamic signals, we randomized the phase distribution of its frequency components (Theiler et al. 1992; Schreiber and Schmitz 1996) (Supplementary Fig. 2, see Methods). The GRU prediction performance showed significantly higher mean CC for fMRI data (*P* = 3.7^*^10^−10^; CC = 0.31 ± 0.01 SEM) than surrogate controls (CC = 0.25 ± 0.01 SEM) (Fig. 2*C*). Figure 2*D* shows the predicted time course from the vessel with the highest prediction score (CC = 0.51, t_lag_ = −1 s) in contrast to the surrogate control signal corresponding to the same vessel (CC = 0.41, t_lag_ = −4 s). This shows that the trained GRU was better at predicting the fMRI signal fluctuations.

In addition, the GRU trained on one rat was used to predict the fMRI fluctuation of five different rats. Figure 2*F* demonstrates trial-specific plots of mean CCs from all vessels in comparison to their surrogate controls (380 vessels from 5 rats), showing significantly higher CC of the fMRI signal (*P* = 6.4^*^10^−24^; CC = 0.29 ± 0.01 SEM) than that of surrogate controls (CC = 0.24 ± 0.01 SEM; Fig. 2*G*). Figure 2*H* shows predicted slow oscillatory time courses of two vessels from different rats based on the trained GRU (v_1_, CC = 0.51, t_lag_ = 0 s; v_2_, CC = 0.52, t_lag_ = 0 s). These results indicate that the fMRI signal fluctuation can be predicted by the trained RNN.

### GRU-Based Single-Vessel fMRI Slow Oscillation Prediction in Awake Human Subjects

**Figure 3:**
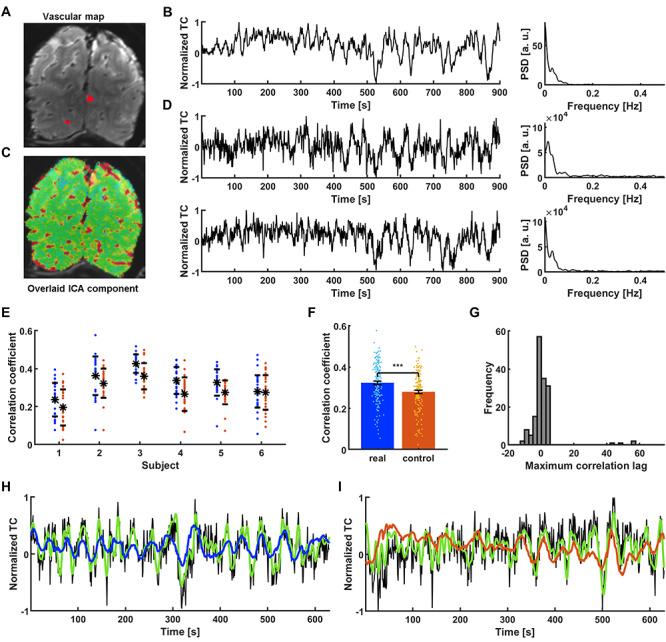
Extraction and prediction of the spontaneous slow fluctuation of human vascular dynamics. (A) The temporal mean of a human single-vessel EPI slice enables the localization of single veins (black dots) in the occipital cortex (red—3 vessel masks; plotted in D. (B) Time course of the slowly changing ICA component shaping vascular dynamics and its PSD. (C) An ICA spatial map highlights the presence of slow fluctuations predominantly in sulcus veins. (D) Two single vessel time courses selected for further processing (marked as red dots in A along with their PSDs. The ICA component is present in the signals, along with individual variations. (E) Prediction scores of all the signals extracted from 6 human subjects (blue dots). Real data are matched with controls for every subject (red dots). (F) Significantly higher mean prediction score of real data (CC = 0.32 ± 0.01 SEM) as compared to controls (CC = 0.28 ± 0.01 SEM; paired-sample t-test, *P* = 1.6^*^10^–13^). (G) Histogram of lags at which the correlation between target outputs and network predictions was the highest. Distribution centered around 0 s (median = 0 s) indicates that the prediction was not simply the filtered input. (H) Prediction plot of the signal that obtained the highest score among all training human vessels (CC = 0.58, t_lag_ = −1 s; black—raw data, green—target, blue—network prediction). (I) Prediction plot of the surrogate control signal created based on the real vascular signal shown in H (CC = 0.50, t_lag_ = 61 s; black—raw data, green—target prediction, red—network output).

As previously reported (Barth and Norris 2007; He et al. 2018), the fMRI signal from sulcus veins of the occipital lobe demonstrated highly correlated slow-oscillatory features (Fig. 3). The vein-specific rs-fMRI signal fluctuations were recorded with high-resolution EPI-fMRI with 840 x 840 μm in-plane resolution and 1.5 mm thickness (Fig. 3*A*, veins are dark dots) and analyzed with ICA. The largest vascular ICA component exhibited slow oscillatory fluctuations in the 0.01–0.1 Hz frequency range (Fig. 3*B*) and its correlation map primarily highlighted the individual sulcus veins in the EPI image (Fig. 3*C*). Figure 3*D* shows raw fMRI time courses from two sulcus veins, demonstrating the vessel-specific time courses and PSDs with varied noise contributions to different veins. Differences between species are visible in the PSDs. A significantly wider range of frequencies contribute strongly to time courses extracted from human vessels compared to rat data (Supplementary Fig. 3, human_FWHM_: 0.031 ± 0.01 Hz; rat_FWHM_: 0.008 ± 0.001 Hz, *P* = 0.001). These results also enable the use of the GRU to encode the slow oscillation based on the vessel-specific fMRI signals from human brains.

In contrast to the multi-trial single-vessel rat fMRI studies, only one trial (15 min) was acquired from each human subject (159 veins from 6 subjects). To perform the supervised training, we designed the 5 + 1 cross-subject validation process (trials from 5 subjects were used for training, and the sixth trial was used for test validation). Specific surrogate control time courses were created based on PSD profiles of fMRI signals acquired from individual veins in the human brain. Using the trained RNN, higher CC values were obtained by predicting slow oscillatory fMRI signals of individual veins compared to their surrogate controls (*P* = 1.6^*^10^−13^; Fig. 3*E*), demonstrating a significantly higher mean CC value for brain dynamic signals (CC = 0.32 ± 0.01 SEM) than for control datasets (CC = 0.28 ± 0.01 SEM) (Fig. 3*F*). Also, the histogram of cross-correlation lag times of the predicted and reference time courses showed a median of the lag time equal to 0, demonstrating the effective prediction (Fig. 3*G*). Figure 3*H* shows an example of a predicted slow oscillatory time course from a human subject based on the trained RNN (CC = 0.58, t_lag_ = −1 s). Figure 3*I* shows the less accurate performance of the matching surrogate control (CC = 0.50, t_lag_ = 61 s). These results demonstrate the GRU-based cross-subject prediction of slow oscillatory fMRI signals.

To inspect the trained network, we applied PCA to the hidden states of the human data-based GRU. Time courses associated with the first component were highly correlated with network inputs. The second component’s signals mostly resembled the generated prediction. Interestingly the third component correlated with the sliding-window score signal (Supplementary Fig. 4*A*). Trajectories of the hidden states in the space defined by the three components seem to be contained on a two dimensional manifold. Different regions of the manifold appear to correspond to the quality of generated predictions (Supplementary Fig. 4*B*). To investigate to which oscillatory features the trained networks were most sensitive, the trained RNNs predicted artificial time courses with a range of peak frequencies and spectral widths (Supplementary Fig. 5*A* and *B*). The predicted spread of the signal spectra preference for GRU_human_ was greater than for GRU_rat_ as shown in the two-dimensional graphs of peak vs. width of the CC distribution (Supplementary Fig. 5*C* and *D*). These species differences may reflect the difference in their rs-fMRI. Interestingly, the harmonic patterns had negative correlations for the preferred frequency, which could be a consequence of the trained RNNs favoring the dominating frequency ranges with the 10 s prediction interval.

### GRU-Based Prediction of the fMRI Slow Oscillation in the Visual Cortex (V1) of HCP Data

**Figure 4:**
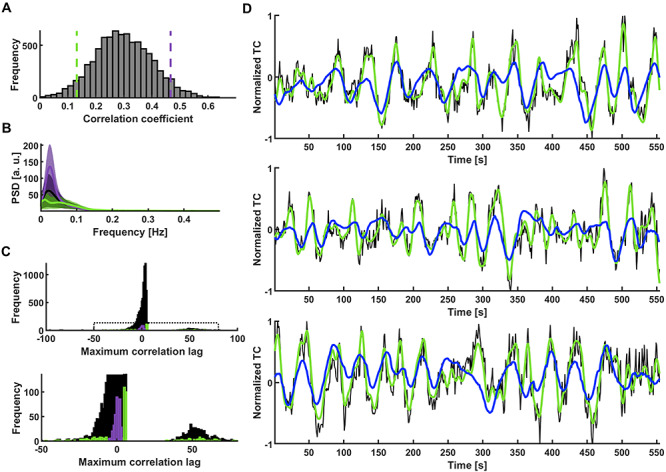
GRU categorization of V1 temporal patterns. (A) Histogram of prediction scores obtained by predicting slow fluctuations of 8024 single-hemisphere V1 ROI signals extracted from HCP data. The used GRU was trained on occipital cortex single-vessel signals of 6 in-house subjects. Green and violet dashed lines mark the bottom and top 5% of correlation coefficients. (B) Mean PSDs of time courses whose predictions obtained the bottom 5% (green) and top 5% (violet) scores. Shaded areas show SD. (C) Histogram of lags at which the correlation between targets and network outputs was the highest. The lags of top 5% of the predictions (violet) are concentrated around 0. The lags of bottom 5% (green) are spread across the highest and lowest lag values. Bottom: Enlarged region marked on the top plot. (D) Predictions of signals with three of the best correlations (CC_1_ = 0.64, _tlag,1_ = −1 s; CC_2_ = 0.62, _tlag, 2_ = 1 s; CC_3_ = 0.62, _tlag, 3_ = −1 s; black—raw data, green—target, blue—network prediction).

Previously, we showed that smoothed single-vessel rs-fMRI correlation maps mimic conventional correlation maps in the human occipital area (He et al. 2018). As shown in the PSD plots (Fig. 3), the vessel-specific fMRI slow oscillation dominates the 0.01–0.1 Hz frequency range. To examine whether the GRU trained by the single-vessel fMRI scheme can be used to predict fMRI slow oscillations of a broader range of datasets, we applied the trained GRU to predict the rs-fMRI signals from the V1 of HCP data (a total of 4012 rs-fMRI sessions; V1 signal extracted from left and right hemispheres separately, yielding 8024 time courses resampled at 1 s TR, details in the Methods section). To examine the predictive capacity of the GRU on each trial of the HCP dataset, the CCs of all prediction trials were plotted in a histogram. The CC distribution resembled a normal distribution centered on 0.29 (median) (Fig. 4*A*).

To specify the rs-fMRI signal temporal dynamics based on the prediction scores, we first selected two clusters of HCP sessions based on the top and bottom 5% CC scores of the overall histogram distribution (Fig 4*A*). The top 5% trials showed much higher power levels than the bottom 5% trials at the 0.01–0.1 Hz frequency range (Fig. 4*B*). The lag time distribution of the top 5% group is centered at zero, unlike the bottom 5% group covering the whole range of lag values (Fig. 4*C*). In particular, many lag values of the poorly predicted sessions show a delay of more than the full wavelength of GRU’s preferred frequency. Figure 4*D* shows three predicted slow oscillatory time courses from the HCP rs-fMRI sessions (top 5% group) (CC_1_ = 0.64, t_lag,1_ = −1 s; CC_2_ = 0.62, t_lag,2_ = 1 s; CC_3_ = 0.62, t_lag, 3_ = −1 s). The predictions of the GRU were dominated by the low-frequency power in the rs-fMRI signals from individual trials.

**Figure 5:**
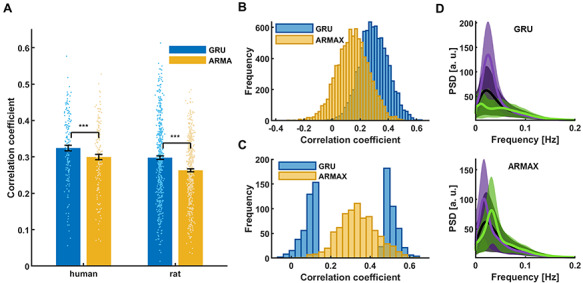
Comparison of different methods’ prediction results. (A) Mean prediction scores of all in-house human and rat vessel signals obtained using the best GRU and ARMAX models. Significantly higher scores (paired-sample t-test, phuman = 8.9^*^10^–9^, prat = 1.9^*^10^–20^) obtained by the RNN than ARMAX in both human (CC_GRU_ = 0.32 ± 0.01; CC_ARMAX_ = 0.30 ± 0.01; mean ± SEM) and rat cases (CC_GRU_ = 0.30 ± 0.01; CC_ARMAX_ = 0.26 ± 0.004; mean ± SEM). (B) GRU and ARMAX histograms of prediction scores of 8024 single-hemisphere V1 ROI signals extracted from HCP data. ARMAX predictions are much worse than those of the GRU. (C) GRU scores of sessions with the 5% best and worst predictions obtained by the both methods. The groups show little overlap. (D) Mean PSDs of time courses whose predictions obtained the bottom 5% (green) and top 5% (violet) scores (top—GRU; bottom—ARMAX). Shaded areas show SD. ARMAX shows less sensitivity to low-frequency power compared to the RNN.

Next, to verify the specific classification of the low-frequency rs-fMRI signal fluctuation by the RNN-based prediction, we compared the GRU predictions with those of ARMAX modeling. The best ARMAX models were found using an exhaustive grid search (see Methods). The RNN prediction scheme presented better performance than ARMAX modeling on our in-house datasets (Human: CC_GRU_ = 0.32 ± 0.01, CC_ARMAX_ = 0.30 ± 0.01; Rat: CC_GRU_ = 0.3 ± 0.01, CC_ARMAX_ = 0.26 ± 0.01; mean ± SEM) (Fig. 5*A*), as well as on the HCP datasets (Fig. 5*B*). In addition, different trials obtained the best and worst scores between the methods (Fig. 5*C*), as the sensitivity to low frequency oscillations was much less pronounced by the ARMAX modeling (Fig. 5*D*). These results confirmed the reliability of the RNN-based rs-fMRI signal predictions.

### RNN-Based Brain State Classification of HCP Data

**Figure 6:**
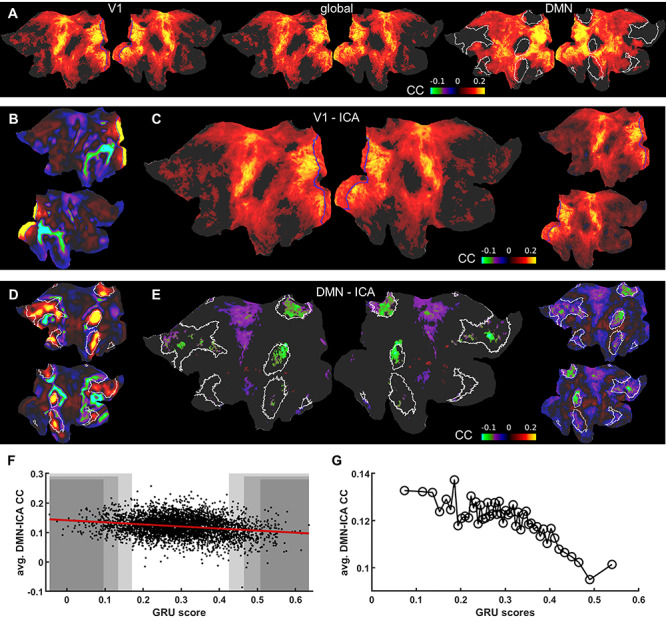
Difference maps for ICA seed-based correlation between well and poorly predicted fMRI sessions. (A) Flattened cortical maps showing the difference between the mean seed-based correlation maps of the 5% “top” and “bottom” groups. The seed signals were: the V1 ROI (left; V1 marked by blue borders), mean global cortical signal (center) and the DMN ROI (right; DMN marked by white borders). (B) V1 ICA component spatial map. V1 ROIs are marked by blue borders. (C) Flattened cortical map showing the difference between the mean seed-based correlation maps of the 5% “top” and “bottom” groups. The time course of the ICA component shown in B served as the seed. V1 ROIs are marked by blue borders. The result resembles the pattern in A obtained by using the V1 ROI as the seed. Nodes in which the difference was insignificant are masked. The same map is shown on the right without the threshold. (D) DMN ICA component spatial map. DMN ROIs are marked by white borders. (E) Flattened cortical map showing the difference between the mean seed-based correlation maps of the 5% “top” and “bottom” groups. The time course of the ICA component shown in D served as the seed. DMN ROIs are marked by white borders. Nodes in which the differences were insignificant are masked (threshold at *P* = 0.05). The intrinsic DMN signals show significantly reduced connectivity with DMN areas. The same map is shown on the right without the threshold. (F) GRU scores of all trials plotted against mean correlations of DMN voxels. Shaded areas cover the top and bottom 2% (dark), 5% and 10% (light) of all scores. (G) GRU scores of all 4012 trials averaged in 2% bins and plotted against mean correlations of DMN voxels.

Here, we investigated the DMN internal correlation, as a brain state marker of the HCP datasets based on the RNN prediction scores. First, we analyzed whole-brain correlation patterns of the HCP dataset, initially focusing on datasets with the top and bottom 5% GRU predictions. Figure 6 shows flattened cortical difference maps of seed-based correlations calculated for the two groups of HCP datasets. First, rs-fMRI time courses from the V1 ROI and the whole cortex (global mean) were used as seeds to calculate voxel-wise correlation maps. The V1 ROI and global mean-based differential maps of the two groups show similar patterns, demonstrating much more synchronized activity across the cortex in the group with top 5% predictions (Fig. 6*A*). Based on the global characteristic of the differences, we computed correlation matrices based on 360 ROIs predefined in the brain atlas (Glasser et al. 2016) as well as 19 subcortical ROIs defined using the Connectome Workbench (Marcus *et al.* 2011). The hippocampus and the brainstem were two subcortical regions which have shown the strongest increase in global correlation (Supplementary Fig. 6*B*). Importantly, we found that the DMN nodes formed a major cluster of regions that did not show the increase across the groups (Supplementary Fig. 6*A*). We followed this result and created the cortical correlation difference map using DMN ROI signals as seeds. Interestingly, although the DMN-ROI also shows higher correlation with the whole brain in the top 5% group, the internal correlations of the DMN-specific nodes do not show significant differences (Fig. 6*A*, the difference between correlations inside and outside of the DMN is significant, *P* = 1.52^*^10^−127^). Representative seed time courses of four subjects from each group are shown in Supplementary Figure 7.

To further investigate the relationship between the internal DMN correlations and the RNN prediction scores, we used ICA component time courses of the V1 area and of the DMN network to analyze the differential maps (Fig. 6*B*-*E*). Figure 6*C* shows that the visual ICA-based map resembled the V1 ROI seed-based differential map, demonstrating a higher correlation feature in the group with the top 5% CC scores. In contrast, as the DMN ICA time courses show a very small global signal content (mean global signal and DMN ICA signal correlation = −0.09+/−0.14 across all trials), significantly reduced internal DMN correlations were observed in the differential map when comparing the top vs. bottom 5% trials (Fig. 6*D* and *E*). To provide a holistic perspective of the RNN prediction scores and brain state relationship, we plotted the internal DMN connectivity as a function of the RNN prediction scores for all individual trials of the HCP datasets. The internal DMN correlations were decreasing as the prediction scores increased (Fig. 6*F* and *G*). This linear relationship of RNN prediction scores and internal DMN activity demonstrates a unique classification scheme to differentiate brain-state dependent rs-fMRI signal fluctuations in the HCP dataset.

**Figure 7:**
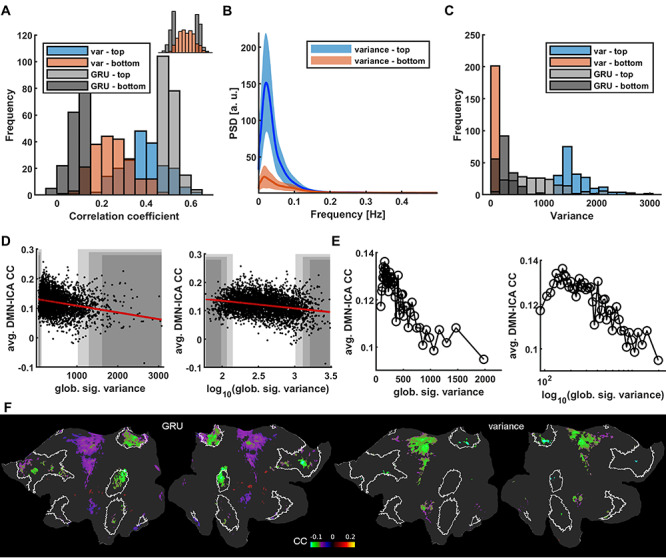
Differences of RNN scores and global signal variance as indicators of intrinsic DMN activity. (A) Prediction score histograms of the 5% best (light gray) and worst (dark gray) predicted sessions contrasted with prediction scores of signals with top 5% highest (blue) and lowest (red) variance. Variance levels are not conclusive of GRU’s performance. Top right: same data with top and bottom groups merged. (B) Mean PSDs of V1 signals from sessions with the top 5% highest (blue) and lowest (red) global signal variance. Shaded areas show standard deviations. The low power difference is more profound than in the RNN score based case (Fig. 4*B*). (C) Histogram of variance-based (red and blue) and GRU-based (gray) group variance values. Variances of signals having high or low prediction scores are distributed across the whole range of variance values. (D) Global signal variance and its logarithm of all trials plotted against mean correlations of DMN voxels. Shaded areas cover the top and bottom 2% (dark), 5% and 10% (light) of all scores. (E) Global signal variance and its logarithm of all 4012 trials averaged in 2% bins and plotted against mean correlations of DMN voxels. At low variance values the DMN correlations decrease breaking the trend. (F) Flattened cortical maps showing the difference between the mean DMN-ICA seed-based correlation maps of the 5% “top” and “bottom” groups based on GRU scores and global signal variance values. DMN ROIs are marked by white borders. Nodes in which the differences were insignificant are masked. The intrinsic DMN signals show significantly reduced connectivity with DMN areas.

Despite the strong linkage to the low frequency power of the rs-fMRI signal, the RNN-based prediction is not simply based on the variance of the rs-fMRI signal fluctuation. By applying a similar analysis scheme, we also classified the HCP datasets based on the variance of the global rs-fMRI signal. Interestingly, the identified groups of sessions with top vs. bottom 5% global signal variance do not show highly distinct GRU prediction CC scores and vice versa (Fig. 7*A*-*C*). In particular, the top and bottom 5% variance groups had much broader CC_GRU_ values and largely overlapped each other in the histogram plot (Fig. 7*A*). Trials with the bottom 5% of GRU predicted CC scores tend to have lower global signal variance, but they overlap with variances of trials with the top 5% scores which cover the whole range of variance values (Fig. 7*C*).

To further understand the issues of the global variance relationship with the internal DMN correlation, we also plotted the DMN correlation values as a function of variance for all trials of the HCP datasets, showing a much more condensed distribution at the low variance ends in the linear scale plot (Fig. 7*D* and *E*). The negative correlation feature can be better depicted in the logarithmic scale plot, but, interestingly, this relationship breaks at low variance values, as for the lowest variance values the DMN correlations decrease (Fig. 7*E*). Thus, the variance-based differential maps of the top vs. bottom 5% trials also show much less DMN node-specific patterns than the RNN-based prediction (Fig. 7*F*). In particular, the RNN prediction-based differential maps highlighted the DMN nodes, e.g., the inferior parietal lobe and posterior cingulate and retrosplenial cortex. In contrast, the variance-based map is much less specific to the internal DMN nodes, but spread more to the somatomotor cortex as demonstrated in the flattened map (Fig. 7*F*). These results indicate that the RNNs trained with vessel-specific rs-fMRI signals encode specific brain state differences, which are not simply explainable by the variance of the rs-fMRI signal fluctuation.

## Discussion

We used the time courses of single-vessel rs-fMRI signals as inputs to train RNN networks to predict the rs-fMRI signal 10 s ahead in both rodents and humans. We also showed that the single-vessel fMRI-based training leads to an RNN encoding specific to low-frequency rs-fMRI signal fluctuations. The trained network was used to analyze HCP datasets with diverse brain states. In particular, it allowed identifying trials, showing unique brain-wide synchrony and to decouple the global signal fluctuations from internal DMN correlations.

We selected the input fMRI time series from individual vessel voxels based on a previously established single-vessel fMRI mapping method (Yu et al. 2016; He et al. 2018; Chen et al. 2019). The BOLD fMRI signal has a direct vascular origin based on the oxy/deoxy-hemoglobin ratio changes (Bandettini et al. 1992; Kwong et al. 1992; Ogawa et al. 1992). The high-resolution single-vessel mapping method allows us to directly extract the venule-dominated BOLD signals with a much higher contrast-to-noise ratio (CNR) than the conventional EPI-fMRI integrating the BOLD signal from both tissue and vessels in large voxels (Menon et al. 1993; Zhang et al. 2012; Yu et al. 2016; He et al. 2018). Although different vessel voxels may present cardiorespiratory noises, e.g., the respiratory volume change (Birn et al. 2006; Birn et al. 2008) or the heartbeat variability (Shmueli et al. 2007; Napadow et al. 2008), a recent simultaneous fMRI and fiber-optic calcium recording study showed a strong correlation of the major ICA vascular component of the rs-fMRI signal fluctuation (Fig. 1) with the calcium signal oscillation (He et al. 2018). Also, these global hemodynamic signal changes are directly correlated with the calcium signal fluctuation through the whole cortex based on optical imaging (Du et al. 2014; Ma et al. 2016; Schwalm et al. 2017; Chen et al. 2020). Thus, the global fMRI signal fluctuation detected from individual vessels represents changing brain states, and not the non-physiological confounding artifacts uniformly distributed through the brain, e.g., the respiration-induced B0 offset (Van de Moortele et al. 2002; Pais-Roldán et al. 2018) or other sources (Murphy et al. 2013; Caballero-Gaudes and Reynolds 2017). In comparison to the voxel-wise or ROI-based time courses from low-resolution EPI images or signals extracted from the biggest major vessels (Tong et al. 2018; Colenbier et al. 2020), the single-vessel rs-fMRI signal provides highly selective datasets for the supervised RNN training to encode brain-state dependent global fMRI signal fluctuations.

The GRU prediction has been analyzed in a great detail from rodent to human rs-fMRI data. The predictions from the trained GRUs vary across vessels as well as across trials. To validate this measurement, we used surrogate controls designed using the IAAFT method (Schreiber and Schmitz 1996). For every vessel, we generated an artificial signal showing a similar frequency power profile (Supplementary Fig. 2) to its corresponding single-vessel rs-fMRI time course, but with randomized phases of complex Fourier components. It has been shown that high-frequency EEG power profiles are highly correlated to the low-frequency EEG signal fluctuation, i.e., phase-amplitude coupling (PAC), in both cortical and subcortical regions for a variety of brain states (Bragin et al. 1995; Steriade et al. 2001; Vanhatalo et al. 2004; Canolty et al. 2006; Fell and Axmacher 2011; Pais-Roldan et al. 2019). This feature has also been used for the correlation analysis of the concurrent EEG and rs-fMRI signal recordings from animals and humans (Goldman et al. 2002; Goense and Logothetis 2008; He et al. 2008; Shmuel and Leopold 2008; Scholvinck et al. 2010; Magri et al. 2012; Murta et al. 2017). Our analysis confirms that the phases of the slow oscillatory rs-fMRI signal carry critical dynamic brain state features (Muller et al. 2018). By randomizing the phases, the surrogate control excludes dynamic brain features but preserves a high similarity in terms of the signal amplitude/power spectral distribution and autocorrelation structure for the verification of the RNN encoding. Also, the spectral characteristics of the GRUs demonstrate different preference maps in terms of the center frequency and the bandwidth depending on the training data from either rat or human data (Supplementary Fig. 5). These training data showed differences in frequency power profiles given the inter-species diversity (de Zwart et al. 2005) and the presence of anesthetics (Du et al. 2014; Ma et al. 2016; Akeju and Brown 2017; Mateo et al. 2017; He et al. 2018; Wu et al. 2019).

The global rs-fMRI signal is a critical confound of correlation analysis with many contributing factors from both physiological and non-physiological sources. In particular, whether the global mean fMRI signal should be removed before the analysis, which can create spurious correlation features, has been debated (Fox *et al.* 2009; Murphy *et al.* 2009; Hahamy *et al.* 2014; Murphy and Fox 2017; Power *et al.* 2017; Billings and Keilholz 2018; Xu *et al.* 2018; Colenbier et al. 2019). Also, the global rs-fMRI signal can over-shadow specific intrinsic RSN features, e.g., the anti-correlation of the DMN and task-positive RSNs (Fox et al. 2005; Hampson et al. 2010; Chen et al. 2017). One intriguing observation based on the RNN predictions is that the internal DMN connectivity is negatively correlated to the prediction scores across trials (Fig. 6*D* and *E*), which is opposite to the positive global correlation observed through the whole brain (Fig. 6*A*-*C*). Both the global signal strength (Wong et al. 2013; Wong et al. 2016) and the intrinsic DMN correlations (De Havas et al. 2012; Ward et al. 2013) have been tied to arousal mediated brain states and the RNN scores reflect a gradient on this arousal axis (Fig. 6*F*-*G*). It is noteworthy that while the global signal variance is also tied to the brain state, its relationship with the internal DMN connectivity is not linear (Fig. 7*D*) and it stops being a good indicator for trials with low variance values. Consequently, the variance-based differential maps show less DMN specificity, but more widespread differences in the somatomotor cortex (Fig. 7*D* and *E*). Thus, the RNN-based approach reveals brain-state specific rs-fMRI signal fluctuations in the HCP datasets.

The contrast between internal DMN correlations and whole brain correlation patterns supports other sources of evidence that the global signals are dissociated from intrinsic brain network correlations (Turchi et al. 2018). Turchi et al. showed that the global rs-fMRI signal fluctuation can be directly modulated by inhibiting the activity of the basal forebrain nuclei, indicating that arousal leads to global rs-fMRI signals (Turchi et al. 2018). Global rs-fMRI signal fluctuations are also correlated with whether the eyes are open or closed (Yang et al. 2007; McAvoy et al. 2008; Bianciardi et al. 2009), pupil dilation (Yellin et al. 2015; Schneider et al. 2016; Pais-Roldán et al. 2020), subject vigilance (Wong et al. 2013; Wong et al. 2016) and dynamic brain state changes that occur during different sleep stages (Fukunaga et al. 2006; Schabus et al. 2007; Horovitz et al. 2008; Spoormaker et al. 2011; Tagliazucchi et al. 2012; Hjelm et al. 2018). Recent fMRI studies with concurrent astrocytic calcium recordings or optogenetics have shown that the rs-fMRI fluctuation can be regulated by the arousal ascending pathway through the central thalamic nuclei and midbrain reticular formation (Wang et al. 2018; Wang et al. 2019), implicating the subcortical regulation of the rs-fMRI signal fluctuation as previously reported from both non-human primate and human rs-fMRI studies (Chang et al. 2016; Liu et al. 2018; Turchi et al. 2018). Importantly, we also observed that the single-vessel rs-fMRI signal is specifically coupled to the global neuronal signal fluctuation (He et al. 2018), which supports our single-vessel RNN training scheme to encode the brain-state specific global rs-fMRI signal fluctuations.

Thus, the RNN-based approach provides a scheme to potentially differentiate brain states based on the global rs-fMRI fluctuation. Given the connection of global signal fluctuations with both neural activity (Scholvinck et al. 2010) and switching state dynamics (Gutierrez-Barragan et al. 2019) this method provides a rs-fMRI analysis approach complementary to previous work on the switching states. Combining the RNN-based fMRI signal prediction with EEG in both animal and human brains will provide direct evidence for the state-dependent features of this predictive approach in future exploration. Another promising direction for future work involves applying the proposed method to study the predictability of slow fluctuations in brain regions other than sensory cortices and to investigate which factors, besides arousal-related brain state changes, drive the predictions. Extending the platform to process whole-brain signals would provide a more synoptic view of regularities present in brain dynamics in different states. Finally, the method could be integrated into a real-time fMRI platform to provide feedback stimuli in a closed-loop control scheme.

## Supplementary Material

Supplementary_Figure_1_bhaa260

Supplementary_Figure_2_bhaa260

Supplementary_Figure_3_bhaa260

Supplementary_Figure_4_bhaa260

Supplementary_Figure_5_bhaa260

Supplementary_Figure_6_bhaa260

Supplementary_Figure_7_bhaa260

supplementary_figure_captions_bhaa260

## References

[ref1] Akeju O , Brown EN. 2017. Neural oscillations demonstrate that general anesthesia and sedative states are neurophysiologically distinct from sleep. Curr Opin Neurobiol. 44:178–185. 28544930 10.1016/j.conb.2017.04.011 PMC5520989

[ref2] Bandettini PA , Wong EC, Hinks RS, Tikofsky RS, Hyde JS. 1992. Time course EPI of human brain function during task activation. Magn Reson Med. 25:390–397. 1614324 10.1002/mrm.1910250220

[ref3] Barnard ST , Pothen A, Simon H. 1995. A spectral algorithm for envelope reduction of sparse matrices. Numerical Linear Algebra with Applications. 2:317–334.

[ref4] Barrett DGT , Morcos AS, Macke JH. 2018. Analyzing biological and artificial neural networks: challenges with opportunities for synergy? Aust Dent J. 10.1016/j.conb.2019.01.007 30785004

[ref5] Barth M , Norris DG. 2007. Very high-resolution three-dimensional functional MRI of the human visual cortex with elimination of large venous vessels. NMR Biomed. 20:477–484. 17405190 10.1002/nbm.1158

[ref6] Beckmann CF , Smith SM. 2004. Probabilistic independent component analysis for functional magnetic resonance imaging. IEEE Trans Med Imaging. 23:137–152. 14964560 10.1109/TMI.2003.822821

[ref7] Behzadi Y , Restom K, Liau J, Liu TT. 2007. A component based noise correction method (CompCor) for BOLD and perfusion based fMRI. Neuroimage. 37:90–101. 17560126 10.1016/j.neuroimage.2007.04.042 PMC2214855

[ref8] Bell AJ , Sejnowski TJ. 1995. An information-maximization approach to blind separation and blind deconvolution. Neural Comput. 7:1129–1159. 7584893 10.1162/neco.1995.7.6.1129

[ref9] Benjamini Y , Hochberg Y. 1995. Controlling the false discovery rate: a practical and powerful approach to multiple testing. J R Stat Soc B Methodol. 57:289–300.

[ref10] Bergstra J , Bardenet M, Bengio Y, Bal KZG. 2011. Algorithms for hyper-parameter optimization. In: Proceedings of the 24th International Conference on Neural Information Processing Systems. Granada, Spain: Curran Associates Inc, pp. 2546–2554.

[ref11] Bergstra J , Yamins D, Cox DD. 2013. Making a science of model search: hyperparameter optimization in hundreds of dimensions for vision architectures. In: Proceedings of the 30th International Conference on International Conference on Machine Learning - Volume 28. Atlanta, GA, USA: JMLR.org, pp. I-115–I-123.

[ref12] Bianciardi M , Fukunaga M, van Gelderen P, Horovitz SG, de Zwart JA, Duyn JH. 2009. Modulation of spontaneous fMRI activity in human visual cortex by behavioral state. Neuroimage. 45:160–168. 19028588 10.1016/j.neuroimage.2008.10.034 PMC2704889

[ref13] Billings J , Keilholz S. 2018. The not-so-global blood oxygen level-dependent signal. Brain Connect. 8:121–128. 29430941 10.1089/brain.2017.0517 PMC5899288

[ref14] Birbaumer N , Elbert T, Canavan AG, Rockstroh B. 1990. Slow potentials of the cerebral cortex and behavior. Physiol Rev. 10.1152/physrev.1990.70.1.1 2404287

[ref15] Birn RM , Diamond JB, Smith MA, Bandettini PA. 2006. Separating respiratory-variation-related fluctuations from neuronal-activity-related fluctuations in fMRI. Neuroimage. 31:1536–1548. 16632379 10.1016/j.neuroimage.2006.02.048

[ref16] Birn RM , Smith MA, Jones TB, Bandettini PA. 2008. The respiration response function: the temporal dynamics of fMRI signal fluctuations related to changes in respiration. Neuroimage. 40:644–654. 18234517 10.1016/j.neuroimage.2007.11.059 PMC2533266

[ref17] Biswal B , Hudetz AG, Yetkin FZ, Haughton VM, Hyde JS. 1997. Hypercapnia reversibly suppresses low-frequency fluctuations in the human motor cortex during rest using echo-planar MRI. J Cereb Blood Flow Metab. 17:301–308. 9119903 10.1097/00004647-199703000-00007

[ref18] Biswal B , Yetkin FZ, Haughton VM, Hyde JS. 1995. Functional connectivity in the motor cortex of resting human brain using echo-planar MRI. Magn Reson Med. 34:537–541. 8524021 10.1002/mrm.1910340409

[ref19] Box GEP . 1976. Time series analysis, forecasting and control. In: Jenkins GM, editor. Rev. San Francisco: Holden-Day.

[ref20] Bragin A , Jando G, Nadasdy Z, Hetke J, Wise K, Buzsaki G. 1995. Gamma (40-100 Hz) oscillation in the hippocampus of the behaving rat. The Journal of Neuroscience: The Official Journal of the Society for Neuroscience. 15:47–60. 7823151 10.1523/JNEUROSCI.15-01-00047.1995 PMC6578273

[ref21] Buzsáki G , Draguhn A. 2004. Neuronal oscillations in cortical networks. Science. 304:1926–1929. 15218136 10.1126/science.1099745

[ref22] Caballero-Gaudes C , Reynolds RC. 2017. Methods for cleaning the BOLD fMRI signal. Neuroimage. 154:128–149. 27956209 10.1016/j.neuroimage.2016.12.018 PMC5466511

[ref23] Calhoun VD , Liu J, Adali T. 2009. A review of group ICA for fMRI data and ICA for joint inference of imaging, genetic, and ERP data. Neuroimage. 45:S163–S172. 19059344 10.1016/j.neuroimage.2008.10.057 PMC2651152

[ref24] Canolty RT , Edwards E, Dalal SS, Soltani M, Nagarajan SS, Kirsch HE, Berger MS, Barbaro NM, Knight RT. 2006. High gamma power is phase-locked to theta oscillations in human neocortex. Science. 313:1626–1628. 16973878 10.1126/science.1128115 PMC2628289

[ref25] Chang C , Glover GH. 2009. Effects of model-based physiological noise correction on default mode network anti-correlations and correlations. Neuroimage. 47:1448–1459. 19446646 10.1016/j.neuroimage.2009.05.012 PMC2995588

[ref26] Chang C , Glover GH. 2010. Time-frequency dynamics of resting-state brain connectivity measured with fMRI. Neuroimage. 50:81–98. 20006716 10.1016/j.neuroimage.2009.12.011 PMC2827259

[ref27] Chang C , Leopold DA, Scholvinck ML, Mandelkow H, Picchioni D, Liu X, Ye FQ, Turchi JN, Duyn JH. 2016. Tracking brain arousal fluctuations with fMRI. Proc Natl Acad Sci U S A. 113:4518–4523. 27051064 10.1073/pnas.1520613113 PMC4843437

[ref28] Chen JE , Glover GH, Greicius MD, Chang C. 2017. Dissociated patterns of anti-correlations with dorsal and ventral default-mode networks at rest. Hum Brain Mapp. 38:2454–2465. 28150892 10.1002/hbm.23532 PMC5385153

[ref29] Chen S , Hu X. 2018. Individual identification using the functional brain fingerprint detected by the recurrent neural network. Brain Connect. 8:197–204. 29634323 10.1089/brain.2017.0561

[ref30] Chen S , Langley J, Chen X, Hu X. 2016. Spatiotemporal Modeling of brain dynamics using resting-state functional magnetic resonance imaging with Gaussian hidden Markov model. Brain Connect. 6:326–334. 27008543 10.1089/brain.2015.0398

[ref31] Chen W , Park K, Pan Y, Koretsky AP, Du C. 2020. Interactions between stimuli-evoked cortical activity and spontaneous low frequency oscillations measured with neuronal calcium. Neuroimage. 210:116554. 31972283 10.1016/j.neuroimage.2020.116554 PMC7418846

[ref32] Chen X , Sobczak F, Chen Y, Jiang Y, Qian C, Lu Z, Ayata C, Logothetis NK, Yu X. 2019. Mapping optogenetically-driven single-vessel fMRI with concurrent neuronal calcium recordings in the rat hippocampus. Nat Commun. 10:5239. 31748553 10.1038/s41467-019-12850-x PMC6868210

[ref33] Cho K , van Merriënboer B, Gulcehre C, Bahdanau D, Bougares F, Schwenk H, editors BY. 2014. Learning Phrase Representations using RNN Encoder–Decoder for Statistical Machine Translation. Doha, Qatar: Association for Computational Linguistics, pp. 1724–1734.

[ref34] Colenbier N , Van de Steen F, Uddin LQ, Poldrack RA, Calhoun VD, Marinazzo D. 2019. Disambiguating the role of blood flow and global signal with partial information decomposition. bioRxiv. 596247. 10.1016/j.neuroimage.2020.116699 32179104

[ref35] Colenbier N , Van de Steen F, Uddin LQ, Poldrack RA, Calhoun VD, Marinazzo D. 2020. Disambiguating the role of blood flow and global signal with partial information decomposition. Neuroimage. 213:116699. 32179104 10.1016/j.neuroimage.2020.116699

[ref36] Cordes D , Haughton VM, Arfanakis K, Carew JD, Turski PA, Moritz CH, Quigley MA, Meyerand ME. 2001. Frequencies contributing to functional connectivity in the cerebral cortex in “resting-state” data. Am J Neuroradiol. 22:1326–1333. 11498421 PMC7975218

[ref37] Cox RW . 1996. AFNI: software for analysis and visualization of functional magnetic resonance Neuroimages. Comput Biomed Res. 29:162–173. 8812068 10.1006/cbmr.1996.0014

[ref38] De Havas JA , Parimal S, Soon CS, Chee MW. 2012. Sleep deprivation reduces default mode network connectivity and anti-correlation during rest and task performance. Neuroimage. 59:1745–1751. 21872664 10.1016/j.neuroimage.2011.08.026

[ref39] de Zwart JA , Silva AC, van Gelderen P, Kellman P, Fukunaga M, Chu R, Koretsky AP, Frank JA, Duyn JH. 2005. Temporal dynamics of the BOLD fMRI impulse response. Neuroimage. 24:667–677. 15652302 10.1016/j.neuroimage.2004.09.013

[ref40] Dezfouli A , Morris R, Ramos FT, Dayan P, Balleine B. 2018. Integrated accounts of behavioral and neuroimaging data using flexible recurrent neural network models, pp. 4228–4237.

[ref41] Du C , Volkow ND, Koretsky AP, Pan Y. 2014. Low-frequency calcium oscillations accompany deoxyhemoglobin oscillations in rat somatosensory cortex. Proc Natl Acad Sci U S A. 111:E4677–E4686. 25313035 10.1073/pnas.1410800111 PMC4217406

[ref42] Elbert T . 1993. Slow cortical potentials reflect the regulation of cortical excitability. In: McCallum WC, editor. Slow Potential Changes in the Human Brain. New York: Springer.

[ref43] Fell J , Axmacher N. 2011. The role of phase synchronization in memory processes. Nat Rev Neurosci. 12:105–118. 21248789 10.1038/nrn2979

[ref44] Filippini N , MacIntosh BJ, Hough MG, Goodwin GM, Frisoni GB, Smith SM, Matthews PM, Beckmann CF, Mackay CE. 2009. Distinct patterns of brain activity in young carriers of the APOE-epsilon4 allele. Proc Natl Acad Sci U S A. 106:7209–7214. 19357304 10.1073/pnas.0811879106 PMC2678478

[ref45] Fox MD , Snyder AZ, Vincent JL, Corbetta M, Van Essen DC, Raichle ME. 2005. The human brain is intrinsically organized into dynamic, anticorrelated functional networks. Proceedings of the National Academy of Sciences of the United States of America. 102:9673. 15976020 10.1073/pnas.0504136102 PMC1157105

[ref46] Fox MD , Zhang D, Snyder AZ, Raichle ME. 2009. The global signal and observed anticorrelated resting state brain networks. J Neurophysiol. 101:3270–3283. 19339462 10.1152/jn.90777.2008 PMC2694109

[ref47] Fukunaga M , Horovitz SG, van Gelderen P, de Zwart JA, Jansma JM, Ikonomidou VN, Chu R, Deckers RH, Leopold DA, Duyn JH. 2006. Large-amplitude, spatially correlated fluctuations in BOLD fMRI signals during extended rest and early sleep stages. Magn Reson Imaging. 24:979–992. 16997067 10.1016/j.mri.2006.04.018

[ref48] Fultz NE , Bonmassar G, Setsompop K, Stickgold RA, Rosen BR, Polimeni JR, Lewis LD. 2019. Coupled electrophysiological, hemodynamic, and cerebrospinal fluid oscillations in human sleep. Science. 366:628–631. 31672896 10.1126/science.aax5440 PMC7309589

[ref49] Gers FA , Schraudolph NN, Schmidhuber R. 2003. Learning precise timing with lstm recurrent networks. J Mach Learn Res. 3:115–143.

[ref50] Glasser MF , Coalson TS, Bijsterbosch JD, Harrison SJ, Harms MP, Anticevic A, Van Essen DC, Smith SM. 2018. Using temporal ICA to selectively remove global noise while preserving global signal in functional MRI data. Neuroimage. 181:692–717. 29753843 10.1016/j.neuroimage.2018.04.076 PMC6237431

[ref51] Glasser MF , Coalson TS, Robinson EC, Hacker CD, Harwell J, Yacoub E, Ugurbil K, Andersson J, Beckmann CF, Jenkinson M, et al. 2016. A multi-modal parcellation of human cerebral cortex. Nature. 536:171–178. 27437579 10.1038/nature18933 PMC4990127

[ref52] Glasser MF , Sotiropoulos SN, Wilson JA, Coalson TS, Fischl B, Andersson JL, Xu J, Jbabdi S, Webster M, Polimeni JR, et al. 2013. The minimal preprocessing pipelines for the human connectome project. Neuroimage. 80:105–124. 23668970 10.1016/j.neuroimage.2013.04.127 PMC3720813

[ref53] Goense JB , Logothetis NK. 2008. Neurophysiology of the BOLD fMRI signal in awake monkeys. Curr Biol. 18:631–640. 18439825 10.1016/j.cub.2008.03.054

[ref54] Golanov EV , Yamamoto S, Reis DJ. 1994. Spontaneous waves of cerebral blood flow associated with a pattern of electrocortical activity. The American Journal of Physiology. 266:R204–R214. 8304543 10.1152/ajpregu.1994.266.1.R204

[ref55] Goldman RI , Stern JM, Engel J Jr, Cohen MS. 2002. Simultaneous EEG and fMRI of the alpha rhythm. Neuroreport. 13:2487–2492. 12499854 10.1097/01.wnr.0000047685.08940.d0 PMC3351136

[ref56] Greicius MD , Krasnow B, Reiss AL, Menon V. 2003. Functional connectivity in the resting brain: a network analysis of the default mode hypothesis. Proc Natl Acad Sci U S A. 100:253–258. 12506194 10.1073/pnas.0135058100 PMC140943

[ref57] Griffanti L , Salimi-Khorshidi G, Beckmann CF, Auerbach EJ, Douaud G, Sexton CE, Zsoldos E, Ebmeier KP, Filippini N, Mackay CE, et al. 2014. ICA-based artefact removal and accelerated fMRI acquisition for improved resting state network imaging. Neuroimage. 95:232–247. 24657355 10.1016/j.neuroimage.2014.03.034 PMC4154346

[ref58] Güçlü U , van Gerven MAJ. 2017. Modeling the dynamics of human brain activity with recurrent neural networks. Front Comput Neurosci. 11:7–7. 28232797 10.3389/fncom.2017.00007 PMC5299026

[ref59] Gutierrez-Barragan D , Basson MA, Panzeri S, Gozzi A. 2019. Infraslow state fluctuations govern spontaneous fMRI network dynamics. Current biology: CB. 29. e2295:2295–2306. 31303490 10.1016/j.cub.2019.06.017 PMC6657681

[ref60] Hahamy A , Calhoun V, Pearlson G, Harel M, Stern N, Attar F, Malach R, Salomon R. 2014. Save the global: global signal connectivity as a tool for studying clinical populations with functional magnetic resonance imaging. Brain Connect. 4:395–403. 24923194 10.1089/brain.2014.0244 PMC4121047

[ref61] Hampson M , Driesen N, Roth JK, Gore JC, Constable RT. 2010. Functional connectivity between task-positive and task-negative brain areas and its relation to working memory performance. Magn Reson Imaging. 28:1051–1057. 20409665 10.1016/j.mri.2010.03.021 PMC2936669

[ref62] Hampson M , Driesen NR, Skudlarski P, Gore JC, Constable RT. 2006. Brain connectivity related to working memory performance. The Journal of Neuroscience: The Official Journal of the Society for Neuroscience. 26:13338–13343. 17182784 10.1523/JNEUROSCI.3408-06.2006 PMC2677699

[ref63] Handwerker DA , Roopchansingh V, Gonzalez-Castillo J, Bandettini PA. 2012. Periodic changes in fMRI connectivity. Neuroimage. 63:1712–1719. 22796990 10.1016/j.neuroimage.2012.06.078 PMC4180175

[ref64] Hansen EC , Battaglia D, Spiegler A, Deco G, Jirsa VK. 2015. Functional connectivity dynamics: modeling the switching behavior of the resting state. Neuroimage. 105:525–535. 25462790 10.1016/j.neuroimage.2014.11.001

[ref65] He BJ , Raichle ME. 2009. The fMRI signal, slow cortical potential and consciousness. Trends Cogn Sci. 13:302–309. 19535283 10.1016/j.tics.2009.04.004 PMC2855786

[ref66] He BJ , Snyder AZ, Zempel JM, Smyth MD, Raichle ME. 2008. Electrophysiological correlates of the brain's intrinsic large-scale functional architecture. Proc Natl Acad Sci U S A. 105:16039–16044. 18843113 10.1073/pnas.0807010105 PMC2564983

[ref67] He Y , Wang M, Chen X, Pohmann R, Polimeni JR, Scheffler K, Rosen BR, Kleinfeld D, Yu X. 2018. Ultra-slow single-vessel BOLD and CBV-based fMRI spatiotemporal dynamics and their correlation with neuronal intracellular calcium signals. Neuron. 97:925–939 e925. 29398359 10.1016/j.neuron.2018.01.025 PMC5845844

[ref68] Hjelm RD , Damaraju E, Cho K, Laufs H, Plis SM, Calhoun VD. 2018. Spatio-temporal dynamics of intrinsic networks in functional magnetic imaging data using recurrent neural networks. Front Neurosci. 12:600. 30294250 10.3389/fnins.2018.00600 PMC6158311

[ref69] Hochreiter S , Schmidhuber J. 1997. Long short-term memory. Neural Comput. 9:1735–1780. 9377276 10.1162/neco.1997.9.8.1735

[ref70] Horovitz SG , Fukunaga M, de Zwart JA, van Gelderen P, Fulton SC, Balkin TJ, Duyn JH. 2008. Low frequency BOLD fluctuations during resting wakefulness and light sleep: a simultaneous EEG-fMRI study. Hum Brain Mapp. 29:671–682. 17598166 10.1002/hbm.20428 PMC6871022

[ref71] Hutchison RM , Womelsdorf T, Allen EA, Bandettini PA, Calhoun VD, Corbetta M, Della Penna S, Duyn JH, Glover GH, Gonzalez-Castillo J, et al. 2013. Dynamic functional connectivity: promise, issues, and interpretations. Neuroimage. 80:360–378. 23707587 10.1016/j.neuroimage.2013.05.079 PMC3807588

[ref72] Hyvarinen A . 1999. Fast and robust fixed-point algorithms for independent component analysis. IEEE Trans Neural Netw. 10:626–634. 18252563 10.1109/72.761722

[ref73] Karahanoglu FI , Van De Ville D. 2015. Transient brain activity disentangles fMRI resting-state dynamics in terms of spatially and temporally overlapping networks. Nat Commun. 6:7751. 26178017 10.1038/ncomms8751 PMC4518303

[ref74] Kleinfeld D , Mitra PP, Helmchen F, Denk W. 1998. Fluctuations and stimulus-induced changes in blood flow observed in individual capillaries in layers 2 through 4 of rat neocortex. Proc Natl Acad Sci U S A. 95:15741–15746. 9861040 10.1073/pnas.95.26.15741 PMC28114

[ref75] Kwong KK , Belliveau JW, Chesler DA, Goldberg IE, Weisskoff RM, Poncelet BP, Kennedy DN, Hoppel BE, Cohen MS, Turner R, et al. 1992. Dynamic magnetic resonance imaging of human brain activity during primary sensory stimulation. Proc Natl Acad Sci U S A. 89:5675–5679. 1608978 10.1073/pnas.89.12.5675 PMC49355

[ref76] Li H , Fan Y. 2018. Brain decoding from functional MRI using long short-term memory recurrent neural networks. Journal. 10.1007/978-3-030-00931-1_37 PMC6180332 30320311

[ref77] Li J , Bolt T, Bzdok D, Nomi JS, Yeo BTT, Spreng RN, Uddin LQ. 2019. Topography and behavioral relevance of the global signal in the human brain. Sci Rep. 9:14286. 31582792 10.1038/s41598-019-50750-8 PMC6776616

[ref78] Liang Z , Liu X, Zhang N. 2015. Dynamic resting state functional connectivity in awake and anesthetized rodents. Neuroimage. 104:89–99. 25315787 10.1016/j.neuroimage.2014.10.013 PMC4252714

[ref79] Linnainmaa S . 1976. Taylor expansion of the accumulated rounding error. BIT Numerical Mathematics. 16:146–160.

[ref80] Liu X , Chang C, Duyn JH. 2013. Decomposition of spontaneous brain activity into distinct fMRI co-activation patterns. Frontiers in systems neuroscience. 7:101. 24550788 10.3389/fnsys.2013.00101 PMC3913885

[ref81] Liu X , de Zwart JA, Scholvinck ML, Chang C, Ye FQ, Leopold DA, Duyn JH. 2018. Subcortical evidence for a contribution of arousal to fMRI studies of brain activity. Nat Commun. 9:395. 29374172 10.1038/s41467-017-02815-3 PMC5786066

[ref82] Liu X , Duyn JH. 2013. Time-varying functional network information extracted from brief instances of spontaneous brain activity. Proc Natl Acad Sci U S A. 110:4392–4397. 23440216 10.1073/pnas.1216856110 PMC3600481

[ref83] Logothetis NK , Pauls J, Augath M, Trinath T, Oeltermann A. 2001. Neurophysiological investigation of the basis of the fMRI signal. Nature. 412:150–157. 11449264 10.1038/35084005

[ref84] Ma Y , Shaik MA, Kozberg MG, Kim SH, Portes JP, Timerman D, Hillman EM. 2016. Resting-state hemodynamics are spatiotemporally coupled to synchronized and symmetric neural activity in excitatory neurons. Proc Natl Acad Sci U S A. 113:E8463–E8471. 27974609 10.1073/pnas.1525369113 PMC5206542

[ref85] Magri C , Schridde U, Murayama Y, Panzeri S, Logothetis NK. 2012. The amplitude and timing of the BOLD signal reflects the relationship between local field potential power at different frequencies. The Journal of Neuroscience: The Official Journal of the Society for Neuroscience. 32:1395–1407. 22279224 10.1523/JNEUROSCI.3985-11.2012 PMC6796252

[ref86] Marcus DS , Harwell J, Olsen T, Hodge M, Glasser MF, Prior F, Jenkinson M, Laumann T, Curtiss SW, Van Essen DC. 2011. Informatics and data mining tools and strategies for the human connectome project. Front Neuroinform. 5:4. 21743807 10.3389/fninf.2011.00004 PMC3127103

[ref87] Masimore B , Kakalios J, Redish AD. 2004. Measuring fundamental frequencies in local field potentials. J Neurosci Methods. 138:97–105. 15325117 10.1016/j.jneumeth.2004.03.014

[ref88] Mateo C , Knutsen PM, Tsai PS, Shih AY, Kleinfeld D. 2017. Entrainment of arteriole vasomotor fluctuations by neural activity is a basis of blood-oxygenation-level-dependent "resting-state" connectivity. Neuron. 96:936–948 e933. 29107517 10.1016/j.neuron.2017.10.012 PMC5851777

[ref89] McAvoy M , Larson-Prior L, Nolan TS, Vaishnavi SN, Raichle ME, d'Avossa G. 2008. Resting states affect spontaneous BOLD oscillations in sensory and paralimbic cortex. J Neurophysiol. 100:922–931. 18509068 10.1152/jn.90426.2008 PMC2525732

[ref90] Mckeown MJ , Makeig S, Brown GG, Jung T-P, Kindermann SS, Bell AJ, Sejnowski TJ. 1998. Analysis of fMRI data by blind separation into independent spatial components. Hum Brain Mapp. 6:160–188. 9673671 10.1002/(SICI)1097-0193(1998)6:3<160::AID-HBM5>3.0.CO;2-1 PMC6873377

[ref91] Menon RS , Ogawa S, Tank DW, Ugurbil K. 1993. Tesla gradient recalled echo characteristics of photic stimulation-induced signal changes in the human primary visual cortex. Magn Reson Med. 30:380–386. 8412612 10.1002/mrm.1910300317

[ref92] Muller L , Chavane F, Reynolds J, Sejnowski TJ. 2018. Cortical travelling waves: mechanisms and computational principles. Nat Rev Neurosci. 19:255–268. 29563572 10.1038/nrn.2018.20 PMC5933075

[ref93] Murphy K , Birn RM, Bandettini PA. 2013. Resting-state fMRI confounds and cleanup. Neuroimage. 80:349–359. 23571418 10.1016/j.neuroimage.2013.04.001 PMC3720818

[ref94] Murphy K , Birn RM, Handwerker DA, Jones TB, Bandettini PA. 2009. The impact of global signal regression on resting state correlations: are anti-correlated networks introduced? Neuroimage. 44:893–905. 18976716 10.1016/j.neuroimage.2008.09.036 PMC2750906

[ref95] Murphy K , Fox MD. 2017. Towards a consensus regarding global signal regression for resting state functional connectivity MRI. Neuroimage. 154:169–173. 27888059 10.1016/j.neuroimage.2016.11.052 PMC5489207

[ref96] Murta T , Chaudhary UJ, Tierney TM, Dias A, Leite M, Carmichael DW, Figueiredo P, Lemieux L. 2017. Phase-amplitude coupling and the BOLD signal: a simultaneous intracranial EEG (icEEG) - fMRI study in humans performing a finger-tapping task. Neuroimage. 146:438–451. 27554531 10.1016/j.neuroimage.2016.08.036 PMC5312786

[ref97] Napadow V , Dhond R, Conti G, Makris N, Brown EN, Barbieri R. 2008. Brain correlates of autonomic modulation: combining heart rate variability with fMRI. Neuroimage. 42:169–177. 18524629 10.1016/j.neuroimage.2008.04.238 PMC2603289

[ref98] Obrig H , Neufang M, Wenzel R, Kohl M, Steinbrink J, Einhaupl K, Villringer A. 2000. Spontaneous low frequency oscillations of cerebral hemodynamics and metabolism in human adults. Neuroimage. 12:623–639. 11112395 10.1006/nimg.2000.0657

[ref99] Ogawa S , Tank DW, Menon R, Ellermann JM, Kim SG, Merkle H, Ugurbil K. 1992. Intrinsic signal changes accompanying sensory stimulation: functional brain mapping with magnetic resonance imaging. Proc Natl Acad Sci U S A. 89:5951–5955. 1631079 10.1073/pnas.89.13.5951 PMC402116

[ref100] Pais-Roldán P , Biswal B, Scheffler K, Yu X. 2018. Identifying Respiration-Related Aliasing Artifacts in the Rodent Resting-State fMRI Frontiers in Neuroscience. 12. 10.3389/fnins.2018.00788 PMC6230988 30455623

[ref101] Pais-Roldan P , Edlow BL, Jiang Y, Stelzer J, Zou M, Yu X. 2019. Multimodal assessment of recovery from coma in a rat model of diffuse brainstem tegmentum injury. Neuroimage. 10.1016/j.neuroimage.2019.01.060 PMC6642798 30708105

[ref102] Pais-Roldán P , Takahashi K, Sobczak F, Chen Y, Zhao X, Zeng H, Jiang Y, Yu X. 2020. Indexing brain state-dependent pupil dynamics with simultaneous fMRI and optical fiber calcium recording. Proc Natl Acad Sci. 201909937. 10.1073/pnas.1909937117 PMC7104268 32139609

[ref103] Pan WJ , Thompson GJ, Magnuson ME, Jaeger D, Keilholz S. 2013. Infraslow LFP correlates to resting-state fMRI BOLD signals. Neuroimage. 74:288–297. 23481462 10.1016/j.neuroimage.2013.02.035 PMC3615090

[ref104] Pascanu R , Mikolov T, Bengio Y. 2013. On the difficulty of training recurrent neural networks. In: Proceedings of the 30th International Conference on International Conference on Machine Learning - Volume 28. Atlanta, GA, USA: JMLR.org, pp. III-1310–III-1318.

[ref105] Paszke A , Gross S, Massa F, Lerer A, Bradbury J, Chanan G, Killeen T, Lin Z, Gimelshein N, Antiga L, et al. 2019. PyTorch: an imperative style, high-performance deep learning. Library. 8024–8035.

[ref106] Plis SM , Hjelm DR, Salakhutdinov R, Allen EA, Bockholt HJ, Long JD, Johnson HJ, Paulsen JS, Turner JA, Calhoun VD. 2014. Deep learning for neuroimaging: a validation study. Front Neurosci. 8. 10.3389/fnins.2014.00229 PMC4138493 25191215

[ref107] Power JD , Plitt M, Laumann TO, Martin A. 2017. Sources and implications of whole-brain fMRI signals in humans. Neuroimage. 146:609–625. 27751941 10.1016/j.neuroimage.2016.09.038 PMC5321814

[ref108] Raichle ME , MacLeod AM, Snyder AZ, Powers WJ, Gusnard DA, Shulman GL. 2001. A default mode of brain function. Proc Natl Acad Sci U S A. 98:676–682. 11209064 10.1073/pnas.98.2.676 PMC14647

[ref109] Robinson EC , Jbabdi S, Glasser MF, Andersson J, Burgess GC, Harms MP, Smith SM, Van Essen DC, Jenkinson M. 2014. MSM: a new flexible framework for multimodal surface matching. Neuroimage. 100:414–426. 24939340 10.1016/j.neuroimage.2014.05.069 PMC4190319

[ref110] Rumelhart DE , Hinton GE, Williams RJ. 1988. Learning representations by back-propagating errors. In: James AA, Edward R, editors. Neurocomputing: foundations of research. MIT Press, pp. 696–699.

[ref111] Salimi-Khorshidi G , Douaud G, Beckmann CF, Glasser MF, Griffanti L, Smith SM. 2014. Automatic denoising of functional MRI data: combining independent component analysis and hierarchical fusion of classifiers. Neuroimage. 90:449–468. 24389422 10.1016/j.neuroimage.2013.11.046 PMC4019210

[ref112] Schabus M , Dang-Vu TT, Albouy G, Balteau E, Boly M, Carrier J, Darsaud A, Degueldre C, Desseilles M, Gais S, et al. 2007. Hemodynamic cerebral correlates of sleep spindles during human non-rapid eye movement sleep. Proc Natl Acad Sci. 104:13164–13169. 17670944 10.1073/pnas.0703084104 PMC1941810

[ref113] Scheffler K , Lehnhardt S. 2003. Principles and applications of balanced SSFP techniques. Eur Radiol. 13:2409–2418. 12928954 10.1007/s00330-003-1957-x

[ref114] Schneider M , Hathway P, Leuchs L, Samann PG, Czisch M, Spoormaker VI. 2016. Spontaneous pupil dilations during the resting state are associated with activation of the salience network. Neuroimage. 139:189–201. 27291493 10.1016/j.neuroimage.2016.06.011

[ref115] Scholvinck ML , Maier A, Ye FQ, Duyn JH, Leopold DA. 2010. Neural basis of global resting-state fMRI activity. Proc Natl Acad Sci U S A. 107:10238–10243. 20439733 10.1073/pnas.0913110107 PMC2890438

[ref116] Schreiber T , Schmitz A. 1996. Improved surrogate data for nonlinearity tests. Phys Rev Lett. 77:635–638. 10062864 10.1103/PhysRevLett.77.635

[ref117] Schwalm M , Schmid F, Wachsmuth L, Backhaus H, Kronfeld A, Aedo Jury F, Prouvot P-H, Fois C, Albers F, van Alst T, et al. 2017. Cortex-wide BOLD fMRI activity reflects locally-recorded slow oscillation-associated calcium waves. Elife. 6:e27602. 28914607 10.7554/eLife.27602 PMC5658067

[ref118] Shmuel A , Leopold DA. 2008. Neuronal correlates of spontaneous fluctuations in fMRI signals in monkey visual cortex: implications for functional connectivity at rest. Hum Brain Mapp. 29:751–761. 18465799 10.1002/hbm.20580 PMC6870786

[ref119] Shmueli K , van Gelderen P, de Zwart JA, Horovitz SG, Fukunaga M, Jansma JM, Duyn JH. 2007. Low-frequency fluctuations in the cardiac rate as a source of variance in the resting-state fMRI BOLD signal. Neuroimage. 38:306–320. 17869543 10.1016/j.neuroimage.2007.07.037 PMC2128785

[ref120] Smith SM , Beckmann CF, Andersson J, Auerbach EJ, Bijsterbosch J, Douaud G, Duff E, Feinberg DA, Griffanti L, Harms MP, et al. 2013. Resting-state fMRI in the human connectome project. Neuroimage. 80:144–168. 23702415 10.1016/j.neuroimage.2013.05.039 PMC3720828

[ref121] Smith SM , Hyvärinen A, Varoquaux G, Miller KL, Beckmann CF. 2014. Group-PCA for very large fMRI datasets. Neuroimage. 101:738–749. 25094018 10.1016/j.neuroimage.2014.07.051 PMC4289914

[ref122] Spoormaker VI , Czisch M, Maquet P, Jancke L. 2011. Large-scale functional brain networks in human non-rapid eye movement sleep: insights from combined electroencephalographic/functional magnetic resonance imaging studies. Philos Transact A Math Phys Eng Sci. 369:3708–3729. 10.1098/rsta.2011.0078 21893524

[ref123] Srivastava N , Hinton G, Krizhevsky A, Sutskever I, Salakhutdinov R. 2014. Dropout: a simple way to prevent neural networks from overfitting. J Mach Learn Res. 15:1929–1958.

[ref124] Steriade M . 2001. Impact of network activities on neuronal properties in Corticothalamic systems. J Neurophysiol. 86:1–39. 11431485 10.1152/jn.2001.86.1.1

[ref125] Steriade M , Timofeev I, Grenier F. 2001. Natural waking and sleep states: a view from inside neocortical neurons. J Neurophysiol. 85:1969–1985. 11353014 10.1152/jn.2001.85.5.1969

[ref126] Tagliazucchi E , von Wegner F, Morzelewski A, Borisov S, Jahnke K, Laufs H. 2012. Automatic sleep staging using fMRI functional connectivity data. Neuroimage. 63:63–72. 22743197 10.1016/j.neuroimage.2012.06.036

[ref127] Theiler J , Eubank S, Longtin A, Galdrikian B, Farmer JD. 1992. Testing for nonlinearity in time series: the method of surrogate data. Phys D. 58:77–94.

[ref128] Tong Y , Hocke LM, Frederick BB. 2019. Low frequency systemic hemodynamic “noise” in resting state BOLD fMRI: characteristics, causes, implications, mitigation strategies, and applications. Front Neurosci. 13. 10.3389/fnins.2019.00787 PMC6702789 31474815

[ref129] Tong Y , Yao J, Chen JJ, Bd F. 2018. The resting-state fMRI arterial signal predicts differential blood transit time through the brain. J Cereb Blood Flow Metab. 39:1148–1160. 29333912 10.1177/0271678X17753329 PMC6547182

[ref130] Turchi J , Chang C, Ye FQ, Russ BE, Yu DK, Cortes CR, Monosov IE, Duyn JH, Leopold DA. 2018. The basal forebrain regulates global resting-state fMRI fluctuations. Neuron. 97 e944:940–952. 29398365 10.1016/j.neuron.2018.01.032 PMC5823771

[ref131] Van de Moortele PF , Pfeuffer J, Glover GH, Ugurbil K, Hu X. 2002. Respiration-induced B0 fluctuations and their spatial distribution in the human brain at 7 tesla. Magn Reson Med. 47:888–895. 11979567 10.1002/mrm.10145

[ref132] Van Essen DC , Ugurbil K, Auerbach E, Barch D, Behrens TE, Bucholz R, Chang A, Chen L, Corbetta M, Curtiss SW, et al. 2012. The human connectome project: a data acquisition perspective. Neuroimage. 62:2222–2231. 22366334 10.1016/j.neuroimage.2012.02.018 PMC3606888

[ref133] Vanhatalo S , Palva JM, Holmes MD, Miller JW, Voipio J, Kaila K. 2004. Infraslow oscillations modulate excitability and interictal epileptic activity in the human cortex during sleep. Proc Natl Acad Sci U S A. 101:5053–5057. 15044698 10.1073/pnas.0305375101 PMC387372

[ref134] Vidaurre D , Smith SM, Woolrich MW. 2017. Brain network dynamics are hierarchically organized in time. Proc Natl Acad Sci. 114:12827–12832. 29087305 10.1073/pnas.1705120114 PMC5715736

[ref135] Wang M , He Y, Sejnowski TJ, Yu X. 2018. Brain-state dependent astrocytic ca(2+) signals are coupled to both positive and negative BOLD-fMRI signals. Proc Natl Acad Sci U S A. 115:E1647–E1656. 29382752 10.1073/pnas.1711692115 PMC5816146

[ref136] Wang X , Leong ATL, Chan RW, Liu Y, Wu EX. 2019. Thalamic low frequency activity facilitates resting-state cortical interhemispheric MRI functional connectivity. Neuroimage. 201:115985. 31299370 10.1016/j.neuroimage.2019.06.063

[ref137] Ward AM , McLaren DG, Schultz AP, Chhatwal J, Boot BP, Hedden T, Sperling RA. 2013. Daytime sleepiness is associated with decreased default mode network connectivity in both young and cognitively intact elderly subjects. Sleep. 36:1609–1615. 24179292 10.5665/sleep.3108 PMC3792376

[ref138] Welch P . 1967. The use of fast Fourier transform for the estimation of power spectra: a method based on time averaging over short, modified periodograms. IEEE Trans Audio Electroacoust. 15:70–73.

[ref139] Wen D , Wei Z, Zhou Y, Li G, Zhang X, Han W. 2018. Deep learning methods to process fMRI data and their application in the diagnosis of cognitive impairment: a brief overview and our opinion. Front Neuroinform. 12:23. 29755334 10.3389/fninf.2018.00023 PMC5932168

[ref140] Whittle P . 1951. Hypothesis testing in time series analysis. Uppsala: Almqvist & Wiksells boktr.

[ref141] Wong CW , DeYoung PN, Liu TT. 2016. Differences in the resting-state fMRI global signal amplitude between the eyes open and eyes closed states are related to changes in EEG vigilance. Neuroimage. 124:24–31. 26327245 10.1016/j.neuroimage.2015.08.053

[ref142] Wong CW , Olafsson V, Tal O, Liu TT. 2013. The amplitude of the resting-state fMRI global signal is related to EEG vigilance measures. Neuroimage. 83:983–990. 23899724 10.1016/j.neuroimage.2013.07.057 PMC3815994

[ref143] Wu G-R , Di Perri C, Charland-Verville V, Martial C, Carrière M, Vanhaudenhuyse A, Laureys S, Marinazzo D. 2019. Modulation of the spontaneous hemodynamic response function across levels of consciousness. Neuroimage. 200:450–459. 31284028 10.1016/j.neuroimage.2019.07.011

[ref144] Xu H , Su J, Qin J, Li M, Zeng LL, Hu D, Shen H. 2018. Impact of global signal regression on characterizing dynamic functional connectivity and brain states. Neuroimage. 173:127–145. 29476914 10.1016/j.neuroimage.2018.02.036

[ref145] Yamins DL , Hong H, Cadieu CF, Solomon EA, Seibert D, DiCarlo JJ. 2014. Performance-optimized hierarchical models predict neural responses in higher visual cortex. Proc Natl Acad Sci U S A. 111:8619–8624. 24812127 10.1073/pnas.1403112111 PMC4060707

[ref146] Yang H , Long XY, Yang Y, Yan H, Zhu CZ, Zhou XP, Zang YF, Gong QY. 2007. Amplitude of low frequency fluctuation within visual areas revealed by resting-state functional MRI. Neuroimage. 36:144–152. 17434757 10.1016/j.neuroimage.2007.01.054

[ref147] Yekutieli D , Benjamini Y, editors. 1997. In: Resampling-based false discovery rate controlling multiple test procedures for correlated test statistics.

[ref148] Yellin D , Berkovich-Ohana A, Malach R. 2015. Coupling between pupil fluctuations and resting-state fMRI uncovers a slow build-up of antagonistic responses in the human cortex. Neuroimage. 106:414–427. 25463449 10.1016/j.neuroimage.2014.11.034

[ref149] Yeo BT , Krienen FM, Sepulcre J, Sabuncu MR, Lashkari D, Hollinshead M, Roffman JL, Smoller JW, Zollei L, Polimeni JR, et al. 2011. The organization of the human cerebral cortex estimated by intrinsic functional connectivity. J Neurophysiol. 106:1125–1165. 21653723 10.1152/jn.00338.2011 PMC3174820

[ref150] Yousefi B , Shin J, Schumacher EH, Keilholz SD. 2018. Quasi-periodic patterns of intrinsic brain activity in individuals and their relationship to global signal. Neuroimage. 167:297–308. 29175200 10.1016/j.neuroimage.2017.11.043 PMC5845807

[ref151] Yu X , He Y, Wang M, Merkle H, Dodd SJ, Silva AC, Koretsky AP. 2016. Sensory and optogenetically driven single-vessel fMRI. Nat Methods. 13:337–340. 26855362 10.1038/nmeth.3765 PMC6298439

[ref152] Yu X , Wang S, Chen DY, Dodd S, Goloshevsky A, Koretsky AP. 2010. 3D mapping of somatotopic reorganization with small animal functional MRI. Neuroimage. 49:1667–1676. 19770051 10.1016/j.neuroimage.2009.09.021 PMC2967485

[ref153] Zhang RC , Lin Y, Yue M, Li Q, Zhang XF, Liu X, Chi H, Chai YF, Wang M. 2012. Effects of ultraviolet-B irradiance on intraspecific competition and facilitation of plants: self-thinning, size inequality, and phenotypic plasticity. PLoS One. 7:e50822. 23226393 10.1371/journal.pone.0050822 PMC3511279

