# Supplementary figures and images for: Predicting the fMRI Signal Fluctuation with Recurrent Neural Networks Trained on Vascular Network Dynamics
Source: Cereb Cortex. 2020 Sep 17;31(2):826–44. doi: 10.1093/cercor/bhaa260 (PMC7906791; doi:10.1093/cercor/bhaa260)

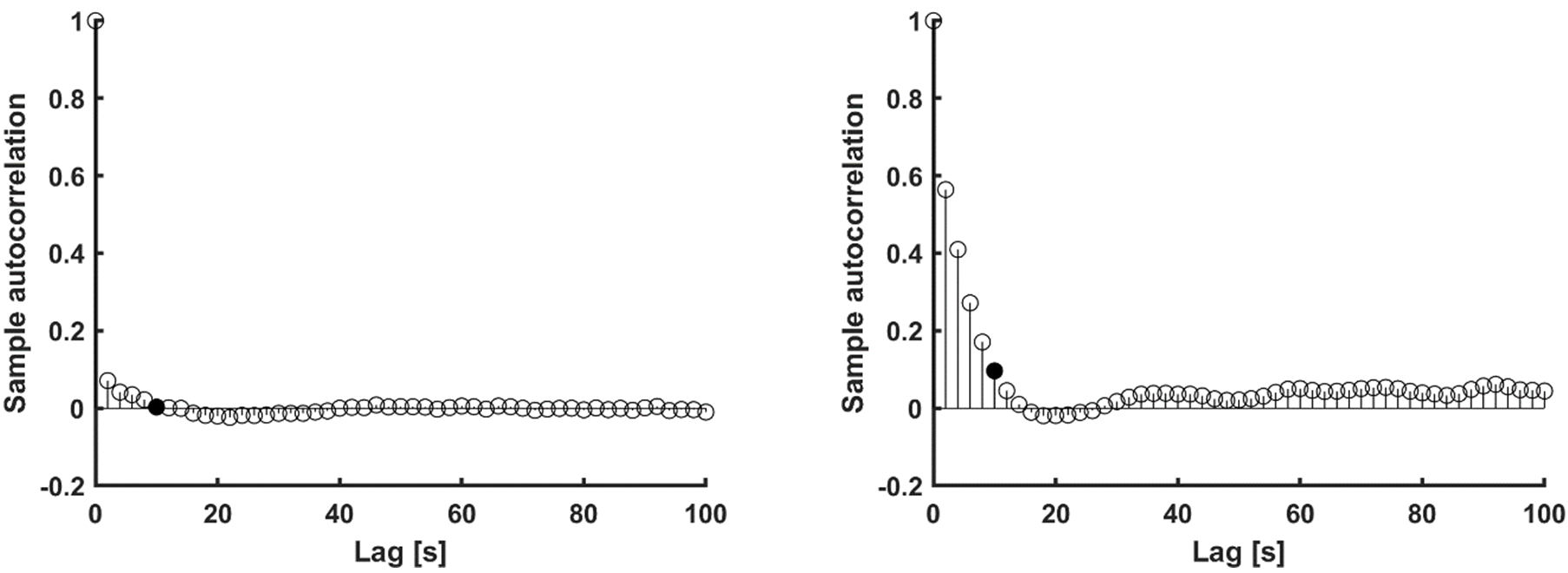

Supplement: Supplementary_Figure_1_bhaa260 [file supplementary_figure_1_bhaa260.png]

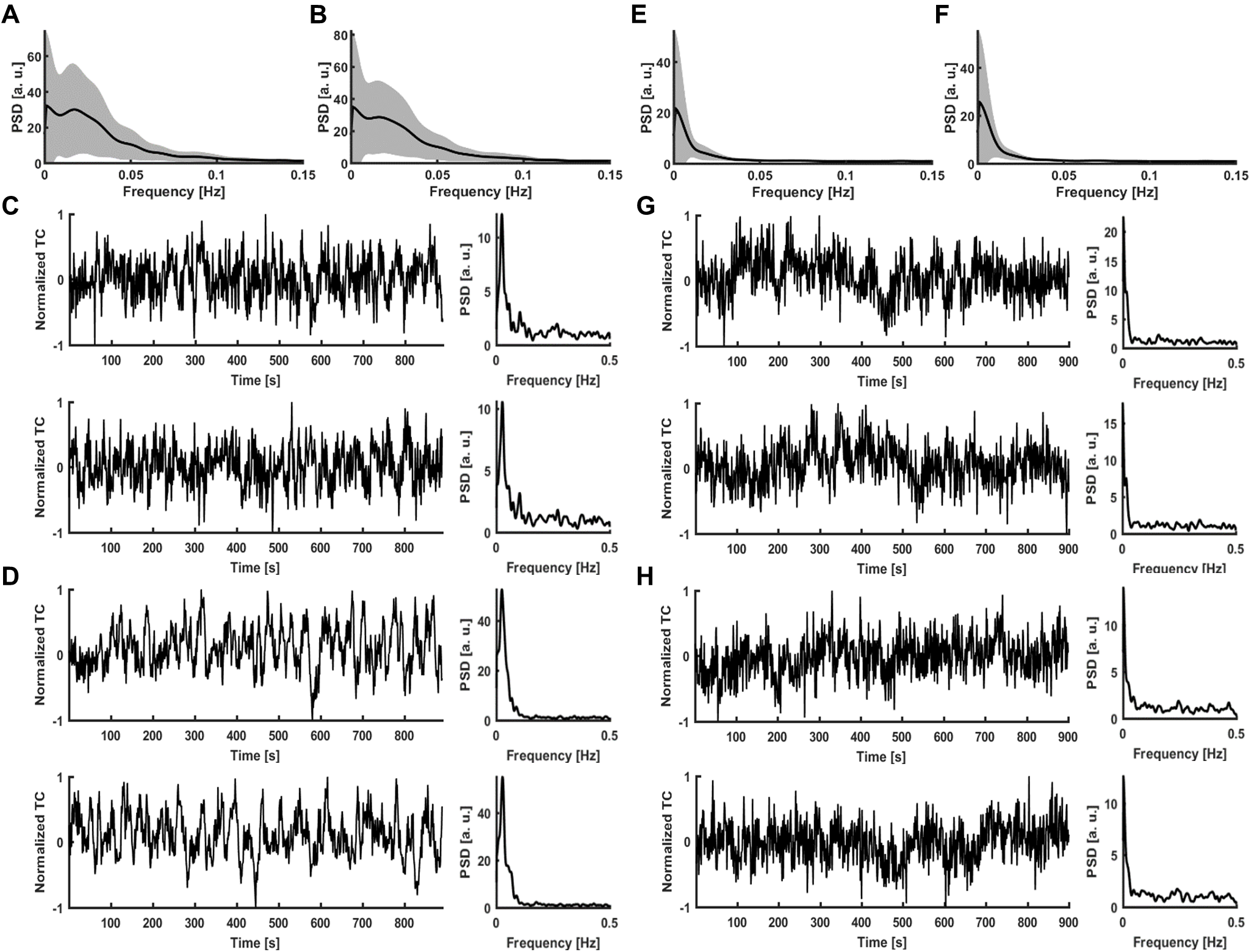

Supplement: Supplementary_Figure_2_bhaa260 [file supplementary_figure_2_bhaa260.png]

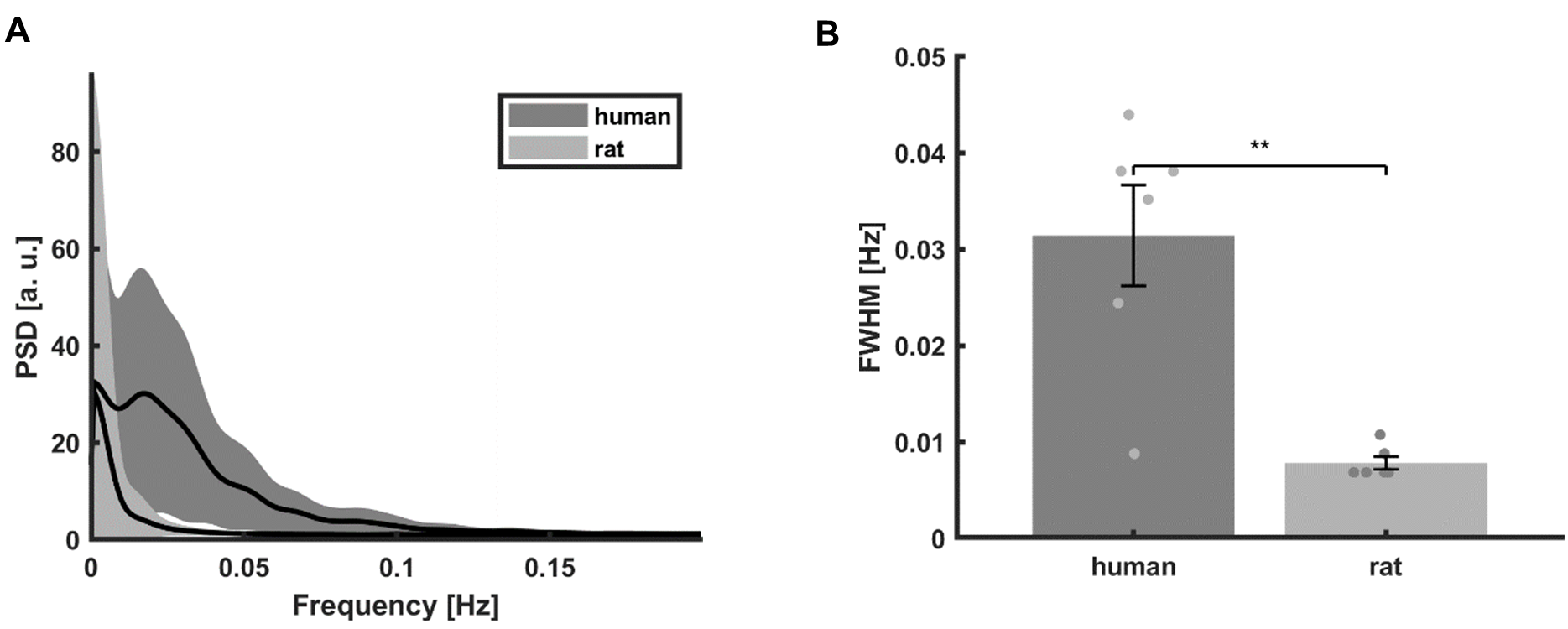

Supplement: Supplementary_Figure_3_bhaa260 [file supplementary_figure_3_bhaa260.png]

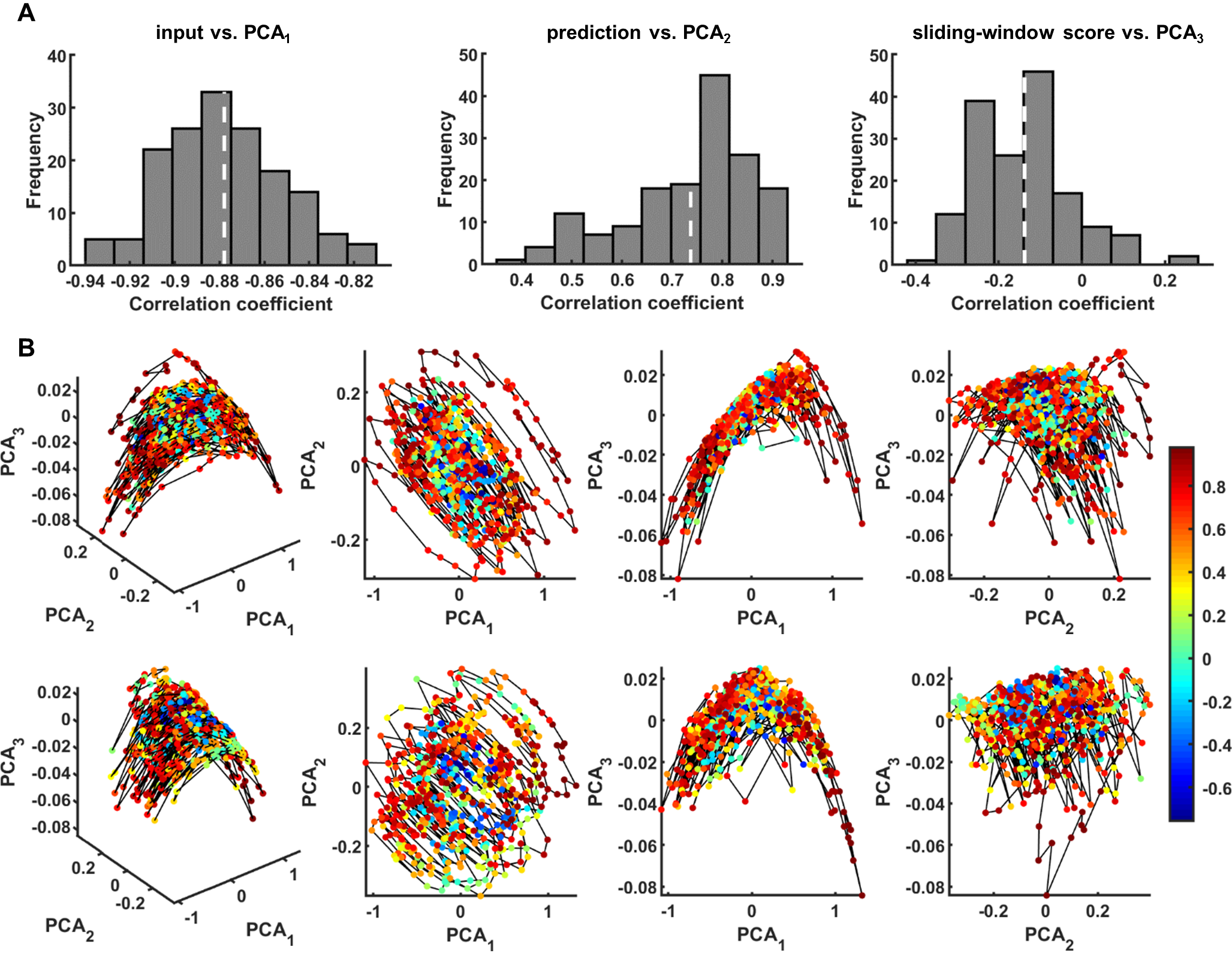

Supplement: Supplementary_Figure_4_bhaa260 [file supplementary_figure_4_bhaa260.png]

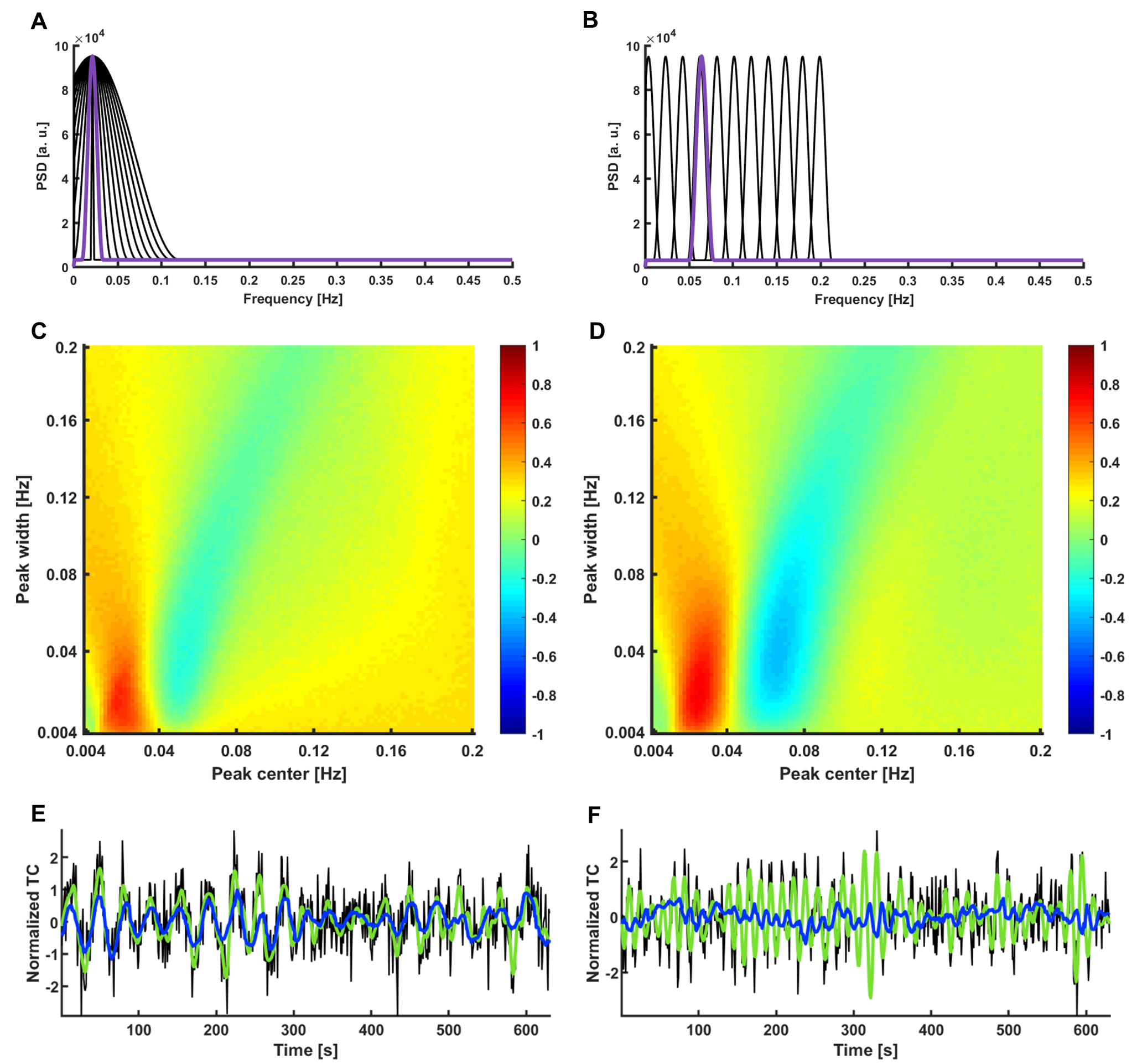

Supplement: Supplementary_Figure_5_bhaa260 [file supplementary_figure_5_bhaa260.png]

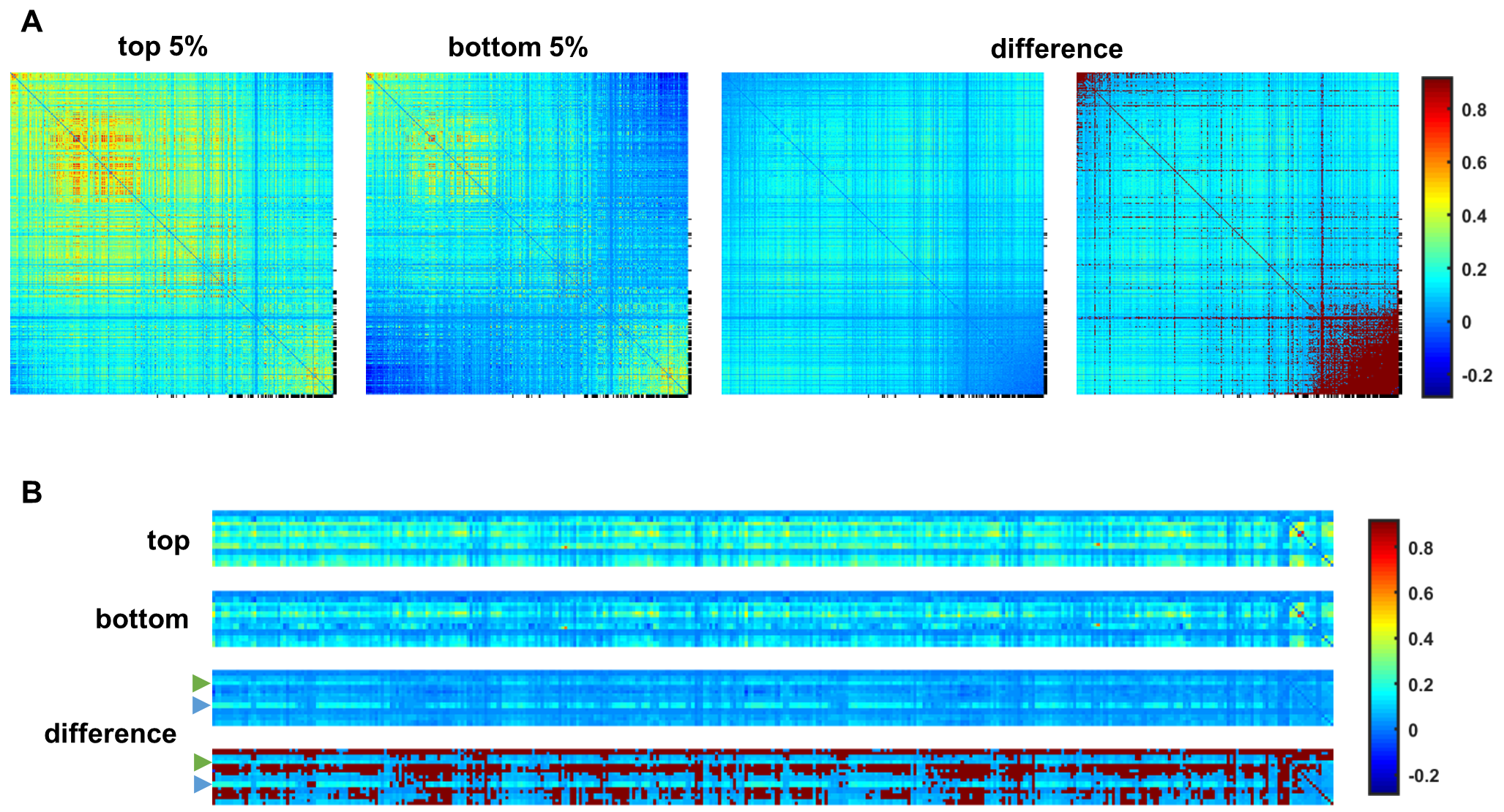

Supplement: Supplementary_Figure_6_bhaa260 [file supplementary_figure_6_bhaa260.png]

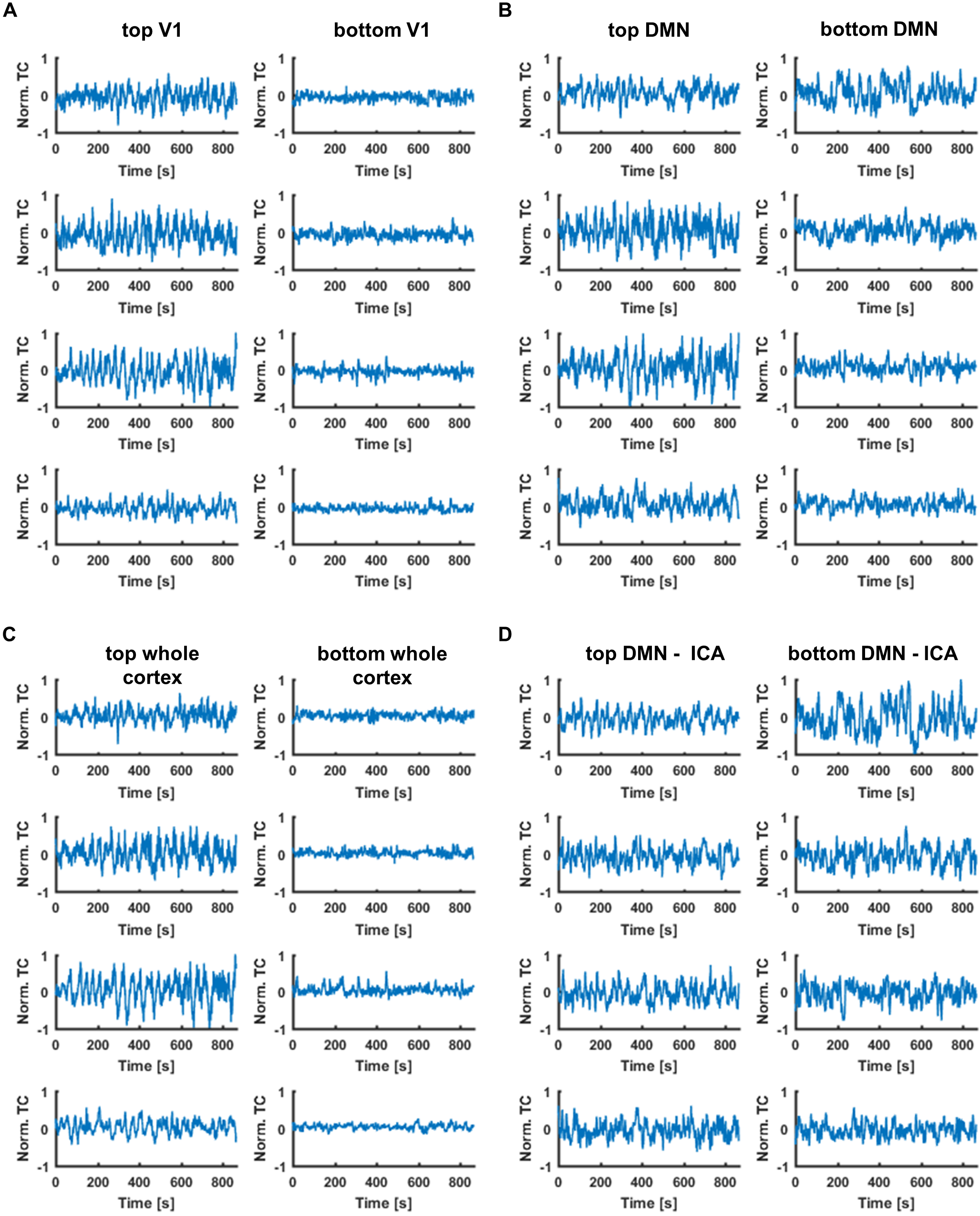

Supplement: Supplementary_Figure_7_bhaa260 [file supplementary_figure_7_bhaa260.png]
