# Supplementary material for: Predicting the fMRI Signal Fluctuation with Recurrent Neural Networks Trained on Vascular Network Dynamics
Source: Cereb Cortex. 2020 Sep 17;31(2):826–44. doi: 10.1093/cercor/bhaa260 (PMC7906791; doi:10.1093/cercor/bhaa260)
Supplement: supplementary_figure_captions_bhaa260 [file supplementary_figure_captions_bhaa260.docx]

**Supplementary Figure 1.** Mean autocorrelation plots of rat (left) and human (right) signals. Values at lags equal to 10 s are marked with a full black dot. For the purpose of this figure, the rat signals have been highpass filtered (0.01 Hz) to remove the autocorrelation resulting from slow drifts.

**Supplementary Figure 2.** Surrogate data. (A) Mean PSD of all human vessel time courses used in the analysis (shaded area – SD). (B) Mean PSD of all surrogate control time courses generated for comparison with human signals (shaded area – SD). (C-D) A real human signal and its PSD (top) matched with the generated surrogate control and its PSD (bottom). (E) Mean PSD of all rat vessel time courses used in the analysis (shaded area – SD). (F) Mean PSD of all surrogate control time courses generated for comparison with rat signals (shaded area – SD). (G-H) A real rat signal and its PSD (top) matched with the generated surrogate control and its PSD (bottom).

**Supplementary Figure 3.** Interspecies PSD and autocorrelation difference. (A) Mean PSDs of all human and rat vessel time courses used in the analysis (shaded areas – SD). (B) Difference of full width at half maximum (FWHM) means of six human subjects‘ mean PSDs (0.031 ± 0.01 SEM) and of six rats’ mean PSDs (0.008 ± 0.001 SEM; paired-sample t-test, p= 0.001).

**Supplementary Figure 4.** PCA of the hidden states of the trained GRU. (A) Histograms of correlations between the signals associated with the first three PCA components and input signals (left; Cc_mean_ = -0.88 ± 0.03 SD), generated predictions (middle; CC_mean_= 0.74 ± 0.13 SD) and (right; CC_mean_= -0.14 ± 0.11 SD) sliding-window score signals. The white dashed line depicts the mean. (B) Trajectories of the hidden states in the space defined by the three components and their 2D projections. Two trials are shown.

**Supplementary Figure 5.** Trained GRU input feature specificity. (A-B) Examples of different artificial PSDs with (A) fixed peak location and varying peak width or (B) fixed peak width and varying peak location used to generate synthetic time courses with specified spectral features. (C-D) Grid displaying the mean prediction scores of time courses generated for each center location – width pair. Values for both the peak width and location were ranging from 0.005-0.2 Hz and were evenly spaced by 0.002 Hz. For each pair 100 signals were generated. Every point on the grid represents their mean prediction score ((C) – rat GRU; (D) – human GRU). (E) Prediction plot of a signal generated from the width (0.023 Hz) and peak location (0.027 Hz) pair best predicted by the human GRU (CC=0.77, t_lag_=0; black – raw data, green – target prediction, blue – network output). (F) Prediction plot of a signal generated from the width (0.045 Hz) and peak location (0.067 Hz) pair worst predicted by the human GRU (CC=-0.40, t_lag_=-15; black – raw data, green – target prediction, blue – network output).

**Supplementary Figure 6.** HCP whole brain functional connectivity. (A) Correlation matrices of 360 cortical ROIs. The ROIs have been arranged based on the order resulting from spectral reordering of the difference (top-bottom) matrix. Most of the cortex displays an increased synchrony in the well predicted sessions. Exceptions are the DMN ROIs (black markers), which despite being more synchronized with the global signal, do not show an increase in internal connectivity. The values on the diagonal have been set to 0. Two difference maps show the same result, but in the right one entries in which the difference was insignificant are masked. (B) Correlation matrices of 19 subcortical ROIs and the cortex. Brainstem (blue arrow) and hippocampus (green arrow) show increased synchrony with the global signal. The rightmost part of the matrices shows internal subcortical correlations. Two difference maps show the same result, but in the lower one entries in which the difference was insignificant are masked.

**Supplementary Figure 7.** Representative seed ROI signals. Time courses extracted from ROIs used as seeds in the connectivity analysis. Signals from the same ROI have been normalized together. 4 sessions from the “top” and “bottom” groups are shown. (A) V1 signals. (B) DMN signals. (C) Global cortical signals. (D) DMN ICA signals.
